# A 6000-year genomic transect in the middle Yellow River

**DOI:** 10.1093/nsr/nwag395

**Published:** 2026-06-26

**Authors:** Rui Wang, Shiwei Li, Guohe Han, Hao Ma, Yukai Lin, Li Jiang, Ziwei Qin, Fulin Ding, Xiaoyan Li, Ting Chen, Wang Shen, Jihua Zhang, Songan Jin, Yuding Zeng, Hongbo Zhai, Yiqiang Lou, Qian Song, Meng Zhang, Xiaomin Yang, Jiajing Zheng, Yu Xu, Tianyou Bai, Le Tao, Haifeng He, Kongyang Zhu, Shaoqing Wen, Li Jin, Xingtao Wei, Caixia Li, Yawei Zhou, Chuan-Chao Wang

**Affiliations:** Ministry of Education Key Laboratory of Contemporary Anthropology, Center for Evolutionary Biology, Department of Anthropology and Human Genetics, School of Life Sciences, Fudan University, Shanghai 200433, China; Henan Provincial Institute of Cultural Heritage and Archaeology, Zhengzhou 450000, China; School of Archaeology and Cultural Heritage, Zhengzhou University, Zhengzhou 450001, China; State Key Laboratory of Cellular Stress Biology, School of Life Sciences, Xiamen University, Xiamen 361102, China; State Key Laboratory of Cellular Stress Biology, School of Life Sciences, Xiamen University, Xiamen 361102, China; National Engineering Laboratory for Forensic Science, Key Laboratory of Forensic Genetics of Ministry of Public Security, Beijing Engineering Research Center of Crime Scene Evidence Examination, Institute of Forensic Science, Beijing 100038, China; State Key Laboratory of Cellular Stress Biology, School of Life Sciences, Xiamen University, Xiamen 361102, China; Henan Provincial Institute of Cultural Heritage and Archaeology, Zhengzhou 450000, China; Henan Provincial Institute of Cultural Heritage and Archaeology, Zhengzhou 450000, China; State Key Laboratory of Cellular Stress Biology, School of Life Sciences, Xiamen University, Xiamen 361102, China; State Key Laboratory of Cellular Stress Biology, School of Life Sciences, Xiamen University, Xiamen 361102, China; School of Archaeology and Cultural Heritage, Zhengzhou University, Zhengzhou 450001, China; School of Archaeology and Cultural Heritage, Zhengzhou University, Zhengzhou 450001, China; State Key Laboratory of Cellular Stress Biology, School of Life Sciences, Xiamen University, Xiamen 361102, China; State Key Laboratory of Cellular Stress Biology, School of Life Sciences, Xiamen University, Xiamen 361102, China; State Key Laboratory of Cellular Stress Biology, School of Life Sciences, Xiamen University, Xiamen 361102, China; Henan Provincial Institute of Cultural Heritage and Archaeology, Zhengzhou 450000, China; Henan Provincial Institute of Cultural Heritage and Archaeology, Zhengzhou 450000, China; Institute of Cultural Heritage, Shandong University, Qingdao 266237, China; State Key Laboratory of Cellular Stress Biology, School of Life Sciences, Xiamen University, Xiamen 361102, China; State Key Laboratory of Cellular Stress Biology, School of Life Sciences, Xiamen University, Xiamen 361102, China; State Key Laboratory of Cellular Stress Biology, School of Life Sciences, Xiamen University, Xiamen 361102, China; Institute of Forensic Science, School of Forensic Medicine and Science, Fudan University, Shanghai 200032, China; State Key Laboratory of Cellular Stress Biology, School of Life Sciences, Xiamen University, Xiamen 361102, China; State Key Laboratory of Cellular Stress Biology, School of Life Sciences, Xiamen University, Xiamen 361102, China; Institute of Archaeological Science, Fudan University, Shanghai 200433, China; Ministry of Education Key Laboratory of Contemporary Anthropology, Center for Evolutionary Biology, Department of Anthropology and Human Genetics, School of Life Sciences, Fudan University, Shanghai 200433, China; Henan Provincial Institute of Cultural Heritage and Archaeology, Zhengzhou 450000, China; National Engineering Laboratory for Forensic Science, Key Laboratory of Forensic Genetics of Ministry of Public Security, Beijing Engineering Research Center of Crime Scene Evidence Examination, Institute of Forensic Science, Beijing 100038, China; School of Archaeology and Cultural Heritage, Zhengzhou University, Zhengzhou 450001, China; Ministry of Education Key Laboratory of Contemporary Anthropology, Center for Evolutionary Biology, Department of Anthropology and Human Genetics, School of Life Sciences, Fudan University, Shanghai 200433, China; State Key Laboratory of Cellular Stress Biology, School of Life Sciences, Xiamen University, Xiamen 361102, China; Institute of Forensic Science, School of Forensic Medicine and Science, Fudan University, Shanghai 200032, China

**Keywords:** ancient genomics, population history, genetic structure

## Abstract

As a pivotal cradle of East Asian civilization, the middle Yellow River region has long been a focal point for studying human cultural and biological evolution. However, its peopling history and kinship practices remain unclear due to a lack of ancient genomic data. In this study, we present 112 newly sequenced ancient genomes from 8 archaeological sites in the middle Yellow River region, dating from 6200 to 100 years ago. Our analyses reveal that all later individuals ultimately traced their primary ancestry to local early populations represented by our newly generated 6200-year-old genomes from western Henan—the earliest genomes from the core area of the Yangshao culture. We also identified two major subsequent demographic transformations. The first was a cultural transition-associated genetic shift around 4500 years ago in western Henan during the Longshan period that may be linked to the spread of Shaanxi-Shanxi populations. This was followed by a two-millennia-long expansion of central Henan-related ancestry that homogenized the genetic landscape across the entire Yellow River basin, independent of significant genetic contributions from the Eurasian Steppe or other adjacent foreign regimes. Beyond demographic history, we provide insights into ancient social structures through two case studies. At the 2500-year-old Shangshihe site, evidence suggests that social status was stably inherited within families and among close biological kin. The society represented by the 2000-year-old Dongxiaoli-Bailong site appears to have placed greater emphasis on the relationships forged through marriage alliances rather than on ancestral lineage inheritance. Collectively, our study illuminates the complex interplay between local continuity and long-distance population connectivity in one of the world’s earliest agricultural heartlands and provides new insights into social structure in Iron Age northern China.

## INTRODUCTION

The Yangshao culture (7000–5000 years ago) originated in the Yellow River (YR) basin and represents one of the earliest, most significant and most extensively studied Neolithic cultures discovered in China. Its core area lies in what is today’s western Henan, southern Shanxi and eastern Shaanxi provinces in the middle YR [1]. Site distribution is particularly dense in the Sanmenxia region of western Henan, especially around Lingbao City, where 193 Neolithic sites have been identified within an area of over 1000 km^2^ centered on Lingbao [2]. Archaeogenetic research has provided novel insights into the demographic history of populations associated with the Yangshao culture in the middle YR, supporting the demic

expansion of the Yangshao culture [3,4]. However, to date, ancient genomic data from the Yangshao cultural period in western Henan remain exceptionally limited; only two genomes have been reported, associated with the early and middle Yangshao periods, respectively [5,6]. The scarcity of ancient genomic data from the core region of the Yangshao culture leaves a critical question unanswered: were the populations within the Yangshao core genetically homogeneous, or did they exhibit population structure that may have persisted through interactions with surrounding cultures?

The fertile lands and strategic location of the Central Plain fostered significant agricultural advancements and population growth, particularly during the Longshan cultural period and China’s early dynastic period. By 4300 years ago, western Henan showed cultural remains associated with the Taosi culture, a subtype of Longshan culture [7]. Our previous ancient genomic research suggested that Longshan cultural era people from western Henan (represented by YR_Yangshaocun_Longshan) [5] did not receive the southern East Asian-related ancestry that previously published contemporaneous people from central Henan did (represented by YR_LN) [6]; instead, they were genetically homogeneous with Shimao_LN, a contemporaneous group from northern Shaanxi associated with the Shimao culture [5,6]. More samples from the Longshan culture in western Henan are needed to determine whether a broader population transition occurred between the Yangshao and Longshan cultural periods in western Henan, or whether this was merely an exception. Furthermore, the Central Plain served as the political, economic and cultural heartland of Chinese civilization for millennia, as evidenced by over 20 dynasties establishing their capitals within its bounds. However, this region also experienced profound shifts driven by factors such as dynastic transitions, internal conflicts and foreign incursions (e.g. from groups like the Rong-di, Xiongnu and Xianbei) [8]. Our previous research reported evidence for high levels of genetic stability within the Central Plain during the specific periods examined, with no significant genetic turnover detected among individuals dating to the Western Zhou dynasty (1046–770 BCE), the Spring and Autumn period (770–476 BCE) and the Tang dynasty (618–907 CE) [9]. Nevertheless, that study contained little data after the Spring and Autumn period and relied on genomic information from a single archaeological site. Consequently, a crucial question remains unresolved: to what extent did cultural interactions between the northern steppe and the Central Plain involve population admixture during key dynastic periods throughout ancient Chinese history?

**Figure 1.**
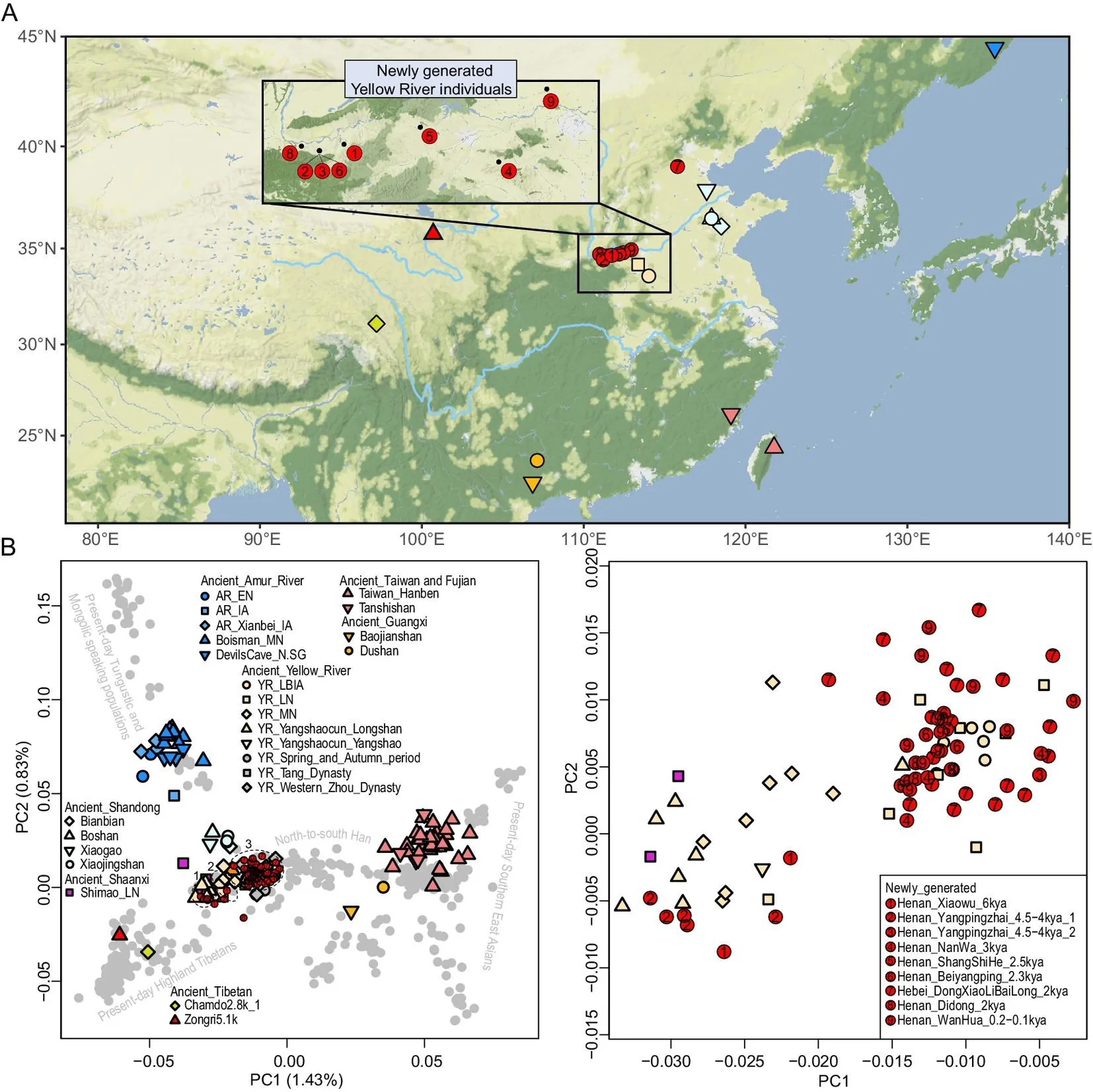
Geographic locations and genetic substructure of our newly generated individuals from 6200–100 years ago in the middle YR. (A) Location of newly generated groups from the middle YR analyzed in the present study and previously published representative populations in East Asia. Symbols as in panel (B). Populations newly sequenced in this study are shown in circled numbers. The first two principal components were constructed from present-day East Eurasians based on the HO dataset (left panel). The right panel is a zoom-in visualization of our newly generated YR ancients and previously published ancients represented by YR_MN, YR_LN, YR_LBIA, YR_Yangshaocun_Yangshao, YR_Yangshaocun_Longshan and Shimao_LN in left panel. The ancient individuals are projected on the PCs constructed by modern East Asian individuals using the ‘lsqproject: YES’ option. Present-day populations are shown in gray, while ancient samples are shown by colored dots. For the group labels, the suffixes represent the archaeological time periods of each group: EN, early Neolithic; MN, middle Neolithic; LN, late Neolithic; BA, Bronze Age; IA, Iron Age. Review drawing number: GS 京(2026)2047号.

In this study, we present genome-wide ancient DNA data from 112 newly reported individuals excavated in Henan and Hebei provinces, northern China (Fig. 1A). These data significantly expand the available genomic record: they include three early Yangshao culture and nine Longshan culture-related genomes from western Henan, and triple the number of individuals from the combined Shang dynasty (∼3000 years ago) through Qing dynasty (∼200–100 years ago) in the Central Plain (Table 1).

**Table 1.** Summary of 112 ancient samples that passed the ancient DNA authenticity test, related to Table S1A.

**Site**	**Location**	**Radiocarbon dating**	**Sample size**
Xiaowu	Lingbao City, Henan, China	6289–6217 cal BP	3
Yangpingzhai	Lingbao City, Henan, China	4575–3988 cal BP	9
Didong	Lingbao City, Henan, China	2343–2146 cal BP	6
Beiyangping	Sanmenxia City, Henan, China	2359–2327 cal BP	13
DongXiaoLiBaiLong	Xiongan New Area, Hebei, China	2093–1927 cal BP	26
ShangShiHe	Yima City, Henan, China	2732–2498 cal BP	38
WanHua	Wuzhi County, Henan, China	291–25 cal BP	13
NanWa	Dengfeng City, Henan, China	3342–3174 cal BP	4

## RESULTS

We generated data from eight archaeological sites in Henan and Hebei provinces in northern China, encompassing an ancient genomic time transect of ∼6000 years (Table S1A). To obtain these data, we extracted DNA from skeletal elements and teeth and generated 119 double-stranded genetic libraries, 67 of which were captured for a targeted set of ∼1.24 million genome-wide single-nucleotide polymorphisms (1240k SNPs). We further evaluated ancient DNA authenticity using two metrics, i.e. ancient DNA damage patterns and contamination estimates based on mitochondrial DNA (mtDNA) and the X chromosome. We found that all individuals showed a typical ancient DNA damage pattern (i.e. high C > T and G > A substitutions at the 5′ and 3′ ends of sequencing reads) (Fig. S1). After removing samples with high contamination rates (>5%) or a low number of SNPs on the 1240k panel (<20 000), 112 individuals were retained (Table S1A).

For subsequent population genetic analyses, we removed first- and second-degree relatives (inferred by READ software [10]) from our newly generated 112 individuals with lower SNP counts (Table S1B). This resulted in 90 individuals retained, with SNP counts ranging from 27 697 to 1 226 898 on the 1240k panel (Table S1A). Finally, we combined these data with previously published ancient and present-day individuals from Eurasia (Table S1C and S1D).

### Overview of the genetic affinities within newly generated individuals from the middle YR

**Figure 2.**
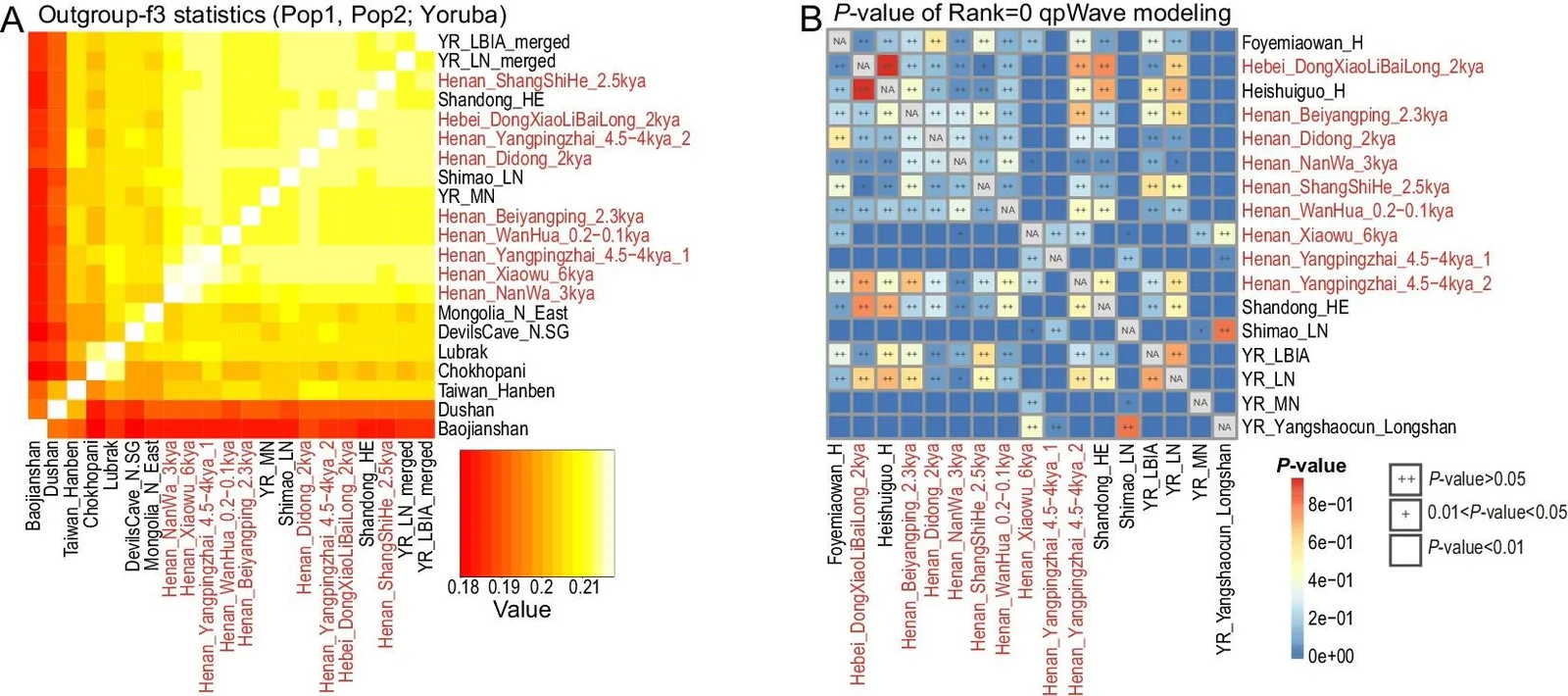
The outgroup-f3 and qpWave analysis for individuals from 6200–100 years ago in the middle YR. (A) Pairwise outgroup-f3 statistics in the form of f3 (popA, popB; Yoruba) to measure the shared genetic drift among ancient East Asians, where yellow indicates higher genetic similarity between pairs. See also Table S2A. (B) Examining whether our newly generated ancient middle YR groups and previously published representative populations in the YR were genetically homogenous with each other. The pairwise qpWave models of Rank = 0 with *P* value > 0.01 suggested that one stream of ancestry from a set of outgroup populations can explain the ancestry of tested populations, in other words, the tested populations are genetically homogenous relative to the base outgroups. See also Table S2B.

To gain an overview of the genetic substructure of all sequenced ancient individuals from the middle YR in comparison with other ancient populations from the broader region and present-day East Asians, we first performed Principal Component Analysis (PCA) (Fig. 1B). We projected all ancient individuals onto the first two principal components calculated using variation in present-day East Asians from the Human Origin (HO) dataset. We found that our newly generated individuals and previously published individuals from the middle YR showed close affinities with each other. These results are also supported by pairwise outgroup-f3 statistics (Fig. 2A), showing that middle YR ancients shared more genetic drift with each other than with all other tested populations.

In the PCA plot, previously published ancient genomes from the middle YR formed three genetic clusters: one related to 5000-year-old Yangshao culture individuals (represented by YR_MN) [6], a second found in 4000-year-old Longshan culture to 3000-year-old individuals from central Henan (represented by YR_LN and YR_LBIA) [6,9,11] and a third represented by 4000-year-old Shimao culture individuals from northern Shaanxi (represented by Shimao_LN) [6] and 4000-year-old Longshan culture individuals from the Yangshaocun site in western Henan (represented by YR_Yangshaocun_Longshan) [5] (Fig. 1B). Two newly generated early Yangshao culture-related individuals from western Henan (labeled Henan_Xiaowu_6kya) clustered with YR_MN (Fig. 1B). Four individuals from the Yangpingzhai site (labeled Henan_Yangpingzhai_4.5–4kya_1) showed a close affinity with YR_MN and YR_Yangshaocun_Longshan (Fig. 1B). Another newly generated Longshan culture-related genome from the Yangpingzhai site (labeled Henan_Yangpingzhai_4.5–4kya_2) clustered with YR_LN (Fig. 1B).

In contrast to the genetic diversity observed among Yangshao and Longshan culture-related individuals from the middle YR, our newly generated 79 unrelated individuals from the same region, dating to 3000 to 100 years ago, did not show significant population substructure in the PCA plot (Fig. 1B). These individuals included 4 Shang dynasty individuals from the NanWa site in Henan (labeled Henan_NanWa_3kya), 35 Spring and Autumn period individuals from the Shangshihe site in Henan (labeled Henan_ShangShiHe_2.5kya), 4 individuals from the Didong site directly dated to 2300 to 2100 years ago (labeled Henan_Didong_2kya), 7 Eastern Zhou dynasty individuals from the Beiyangping site in Henan (labeled Henan_Beiyangping_2.3kya), 20 Han dynasty individuals from the Dongxiaoli-Bailong site in Hebei (labeled Hebei_DongXiaoLiBaiLong_2kya) and 12 Ming and Qing dynasty individuals from the WanHua site in Henan (labeled Henan_WanHua_0.2–0.1kya). It should be noted that one Didong individual deviated from the main cluster, largely due to its ultralow coverage in the HO dataset. For subsequent quantitative analyses, we grouped individuals based on their archaeological sites and genetic classifications.

### Early Yangshao culture-related people from the core region of the Yangshao culture

We newly generated two unrelated early Yangshao culture-related genomes from the Xiaowu site (labeled Henan_Xiaowu_6kya), which represent the earliest Yangshao culture-related genomes from western Henan in the middle YR to date. To quantify the genetic relationship between our newly generated early Yangshao individuals and the late Yangshao culture-related individuals (represented by 5200-year-old YR_MN individuals from the Wanggou site), we performed outgroup-f3 statistics in the form of f3 (Yoruba; Target, Reference) (Fig. S2 and Table S2A). This analysis showed that the early Yangshao period Xiaowu and the late Yangshao period YR_MN shared high levels of genetic drift with each other, suggesting a high degree of genetic continuity among Yangshao culture-related people in the middle YR.

To assess whether late Yangshao culture-related people in the middle YR were the direct descendants of early Yangshao culture-related individuals from the core region of the Yangshao culture, we formally investigated this hypothesis with pairwise qpWave analysis. We found that qpWave modeling showed that a single source, i.e. Henan_Xiaowu_6kya, could successfully explain the genetic makeup of late Yangshao culture people from the middle YR (Fig. 2B and Table S2B). We also calculated f4 statistics in the form of f4 (Yoruba, X; Henan_Xiaowu_6kya, YR_MN) to test for potential differential affinities of early Yangshao and late Yangshao to 233 ancient Eurasians available in our dataset (Fig. S3 and Table S2C). If the late Yangshao culture people were unadmixed descendants of the early Yangshao culture people, we would expect that all f4 statistics would be non-significant. We found that almost all f4 statistics produced non-significant values, suggesting that Henan_Xiaowu_6kya and YR_MN were similarly related to the populations to which they were compared. The main exceptions were those cases where Henan_Xiaowu_6kya shared greater genetic similarity with some ancient Tibetans relative to YR_MN, e.g. f4 (Yoruba, Shannan3k/Nagqu2.7k/Mebrak [12]; Henan_Xiaowu_6kya, YR_MN) < 0 (−3.465 < Z-scores< −2.547) (Fig. S3 and Table S2C). We cannot exclude the possibility that the early Yangshao population had an additional genetic affinity with some ancient Tibetans.

### Both Shimao_LN and YR_LN-related ancestral lineages were observed in 4000-year-old Longshan culture-related people from western Henan

To assess whether Yangshao and Longshan culture individuals showed evidence of genetic continuity through time in western Henan, we computed f3 (Yoruba; Henan_Yangpingzhai_4.5–4kya_1/Henan_Yangpingzhai_4.5–4kya_2, Reference) (Fig. S2 and Table S2A). We observed that both Henan_Yangpingzhai_4.5–4kya_1 and Henan_Yangpingzhai_4.5–4kya_2 showed close affinities with local early Yangshao culture-related people (represented by Henan_Xiaowu_6kya) and YR_MN. These results support genetic continuity between early Yangshao and Longshan culture groups in western Henan.

We then attempted to characterize the ancestral sources of Henan_Yangpingzhai_4.5–4kya_1 and Henan_Yangpingzhai_4.5–4kya_2. We used YR_MN as the reference population, as its shared high genetic drift with the Henan_Yangpingzhai_4.5–4kya people and had a low missing rate and large sample size. Pairwise qpWave analysis rejected the models in which Henan_Yangpingzhai_4.5–4kya_1 and Henan_Yangpingzhai_4.5–4kya_2 were unadmixed descendants of YR_MN (Fig. 2B and Table S2B). F4 tests in the form of f4 (Yoruba, X; Henan_Yangpingzhai_4.5–4kya_1, YR_MN) also revealed that Henan_Yangpingzhai_4.5–4kya_1 had significantly higher affinity with ancient Northeast Asians and ancient Tibetans than YR_MN, similar to the pattern observed in YR_Yangshaocun_Longshan (Fig. S4 and Table S2C). Moreover, in the outgroup-f3 statistics, Henan_Yangpingzhai_4.5–4kya_1 shared high genetic drift with previously published Yangshaocun Longshan culture-related people, who were genetically homogeneous with Shimao_LN (Fig. S2 and Table S2A). In pairwise qpWave analysis, we observed that the ancestral composition of Henan_Yangpingzhai_4.5–4kya_1 could be adequately explained by YR_Yangshaocun_Longshan/Shimao_LN (Fig. 2B and Table S2B). The presence of a Shimao_LN-related genetic profile in both YR_Yangshaocun_Longshan and Henan_Yangpingzhai_4.5–4kya_1 suggests a broader genetic connection between northern Shaanxi and western Henan. We then focused on exploring the genetic composition of Henan_Yangpingzhai_4.5–4kya_2. In f4 statistics of the form of f4 (Yoruba, X; Henan_Yangpingzhai_4.5–4kya_2, YR_MN), we observed that, compared with YR_MN, Henan_Yangpingzhai_4.5–4kya_2 shared more alleles with southern East Asian-related groups (represented by Taiwan_Hanben, Z-score= −2.376) (Fig. S5 and Table S2C). Two-source admixture modeling performed with qpAdm revealed that models using middle YR-related ancestry (represented by 5000-year-old YR_MN) and southern East Asian-related ancestry (represented by 5400-year-old Songze_FuQuanShan, 5300-year-old Liangzhu_MaQiao and 6000-year-old Daxi_DaXi_2 from middle and lower Yangtze River [3]; 8300–6400 years ago Dushan and Baojianshan from inland southern East Asia [13]; 4300-year-old Tanshishan, 4500-year-old Xitoucun [14] and 1500-year-old Taiwan_Hanben [15] from coastal East Asia) are feasible for Henan_Yangpingzhai_4.5–4kya_2 (Table S2E). With this model, we estimated that between 20.6% ± 4.8% and 65.5% ± 12.3% of the ancestry in Henan_Yangpingzhai_4.5–4kya_2 is southern East Asian-related (represented by Taiwan_Hanben, Liangzhu_MaQiao and Songze_FuQuanShan). qpWave analysis supports genetic homogeneity between Henan_Yangpingzhai_4.5–4kya_2 and YR_LN (Fig. 2B and Table S2B).

Collectively, these findings reveal the presence of two genetically distinguishable lineages—initially described in YR_LN from central Henan and Shimao_LN from northern Shaanxi, respectively—in Longshan culture individuals from western Henan.

### A highly connected gene pool across the Yellow River during China’s dynastic period

We next investigated the demographic history of the Central Plain during China’s dynastic period, starting at around 3500 years ago with the establishment of the Shang dynasty. We analyzed data from 82 unrelated individuals from Hebei and Henan with sufficient SNP coverage on the 1240k panel, originating from six sites radiocarbon dated to 3300 to 100 years ago. Focusing first on PCA, we found that, in contrast to our newly sequenced Neolithic samples from Henan, all 3000 to 100 years ago individuals from Henan and Hebei did not display population substructure (Fig. 1B). They all showed genetic affinity with a previously characterized YR_LN-related ancestry. Outgroup-f3 statistics also support the close relationship among these 3000 to 100 years ago groups in the Central Plain (Fig. 2A). These results suggest an ancestral shift and a decrease in the complexity of the genetic composition of the Central Plain following imperial expansion.

We next explored whether these groups were genetically homogeneous using formal tests. There is well-documented evidence for connections between the capital of the Shang dynasty in the Central Plain and other regions of ancient China. For example, metal resources from the middle Yangtze River, hard pottery and primitive porcelain from the lower Yangtze River, salt from the eastern coast, domestic horse from the northern steppe and even seashells and tortoise shells from as far as the South China Sea all converged on the capital of the Shang dynasty [16–18]. Whether the flow of resources was accompanied by large-scale population migrations that shaped the genetic composition of the Shang capital remains unknown. Comparing the genomes related to Longshan and Erlitou cultures [11] and our newly generated five unrelated genomes from the Shang dynasty in central Henan using f4 (Yoruba, X; Henan_NanWa_Shang_Dynasty, YR_LN/middle_YR_Wangchenggang_Erlitou), we observed that most f4 values were non-significant (Fig. S6 and Table S2C). This indicates that, compared with local preceding Longshan and Erlitou people from central Henan, Shang dynasty people did not receive additional genetic contributions from other lineages.

According to historical records, there were frequent incursions by non-Han groups into the Central Plain, such as the Rong-Di during the Eastern Zhou dynasty and the Xiongnu during the Han dynasty. Therefore, we next focused on assessing whether significant genetic introgression from these nomadic populations into the Central Plain gene pool occurred during these periods. We used ancient genomes associated with the Qijia culture from northwestern China (represented by Upper_YR_LN [6]), ancient Xianbei genomes from northeastern China (represented by AR_Xianbei_IA [6]) and ancient Xiongnu genomes from the Mongolia Plateau (represented by Mongolia_Xiongnu.SG, earlyXiongnu_west, earlyXiongnu_rest [19,20]) to represent the genetic components of the Rong-Di, Xianbei and Xiongnu, respectively. If our newly generated Eastern Zhou dynasty group (labeled Henan_ShangShiHe_2.5kya and Henan_Beiyangping_2.3kya) and Han dynasty group (labeled Hebei_DongXiaoLiBaiLong_2kya) received significant Rong-Di/Xianbei/Xiongnu-related ancestry compared with YR_LN, we would expect negative values in f4 statistics of the form f4 (Yoruba, X; studied group, YR_LN) (Fig. S6 and Table S2C). However, we observed that most f4 statistics produced non-significant values (|Z-score| < 3). These results reveal that, compared with diverse ancient Eurasians, each 3000 to 2000 years ago Henan group and 2000 years ago Hebei group formed a clade with YR_LN, the dominant genetic makeup in the Central Plain during the Longshan cultural period. Pairwise qpWave analysis also supports that each 3000 to 2000 years ago Henan group and 2000 years ago Hebei group is consistent with being a clade relative to a list of representative Eurasian lineages, further supporting the long-term genetic stability of the Central Plain (Fig. 2B and Table S2B). Moreover, pairwise qpWave analysis also supports genetic homogeneity among groups from our newly generated Central Plain genomes in the middle YR, historical-era genomes from the lower YR (represented by Shandong_HE) [21] and the upper YR (represented by Heishuiguo_H and Foyemiaowan_H) [22] (Fig. 2B and Table S2B).

It should be noted that our newly sequenced genomes related to Henan_Beiyangping_2.3kya and reanalyzed published 3000-year-old YR_Western_Zhou_Dynasty (labeled YR_LBIA_merged) were all from the same region as the 4000-year-old Yangpingzhai site. We found that Henan_Beiyangping_2.3kya and previously published 3000-year-old YR_Western_Zhou_Dynasty clustered together in PCA, suggesting that they may represent the main ancestry in western Henan during 3000–2300 years ago. F4 statistics further suggest that Henan_Beiyangping_2.3kya and YR_Western_Zhou_Dynasty shared more alleles with southern East Asian-related people compared with local 4000-year-old genomes (represented by YR_Yangshaocun_Longshan and Henan_Yangpingzhai_4.5–4kya_1) (Table S2C and Fig. S7). This suggests a disruption of genetic continuity in western Henan, attributable to an influx of southern East Asian-related gene flow. A parsimonious explanation for this increase in southern East Asian-related ancestry is substantial eastward migration of populations associated with YR_LN-related ancestry, who also share more alleles with southern East Asian-related people compared with Henan_Yangpingzhai_4.5–4kya_1-related ancestry (Fig. S4 and Table S2C). This model is supported by pairwise qpWave analysis, which fails to reject the genetic homogeneity between YR_LN and Henan_Beiyangping_2.3kya/YR_Western_Zhou_Dynasty (Fig. 2B and Table S2B).

Our previous study obtained only one available genome from the Ming and Qing dynasties, and this individual was coincidentally a genetic outlier, as it carried substantial southern East Asian-related ancestry compared with local preceding people. In this study, we newly generated 12 unrelated individuals dating to the Ming and Qing dynasties (labeled Henan_WanHua_0.2–0.1kya). We found that these individuals were genetically homogeneous with each other. Moreover, the Henan_WanHua_0.2–0.1kya group could be modeled as unadmixed descendants of local preceding people (represented by YR_LN, middle_YR_Wangchenggang_Erlitou, Henan_NanWa_3kya, Henan_ShangShiHe_2.5kya and Henan_Beiyangping_2.3kya) (Fig. 2B and Table S2B). These results support long-term genetic stability in the Central Plain.

### Kinship practices at the Shangshihe and Dongxiaoli-Bailong sites in the Central Plain

Leveraging extensive sampling and rich archaeological information from the Shangshihe and Dongxiaoli-Bailong cemeteries in the Central Plain [23–25], we recovered ancient DNA from multiple individuals to investigate their kinship practices. READ [10] was used to identify first- and second-degree genetic relationships (Table S1C), KIN [26] was used to detect relatives up to third-degree and to distinguish between sibling and parent–child pairs (Table S1D), and ancIBD [27] was applied to uncover biological relatedness up to the sixth degree (Table S1E).

#### Genetic relatedness estimation for the Shangshihe site

The Shangshihe site, located in Yima City, Henan Province in northern China, is an archaeological site dating to the Spring and Autumn period (770–403 BCE) [28]. Multiple lines of archaeological evidence indicate the presence of social stratification at the Shangshihe site [25]. Based on tomb orientation, the tombs can be classified into two types: north–south oriented tombs and east–west oriented tombs. Individuals interred in north–south orientated tombs are considered to have held higher social status than those buried in east–west oriented tombs, leading to the hypothesis that the Shangshihe cemetery was divided into two socially stratified groups distinguished by tomb orientation [25]. Nevertheless, key questions remain concerning how genetic ancestry and biological kinship shaped the social stratification observed at the Shangshihe cemetery.

In this study, we successfully recovered genome-wide data from 38 individuals from the Shangshihe cemetery (Table S1A and S1F). Among the 15 pairs of related individuals (whose closest relationships ranged from first- to sixth-degree relatives), 10 pairs were buried in the tombs that shared the same tomb orientation—eight in north–south and 2 in east–west oriented tombs. This suggests that social status was likely stably inherited within families and among close biological kin. The remaining five pairs consisted of individuals with second-degree or higher relationships who were buried in differently oriented tombs. Notably, four of these five close kinship ties were either female–female or female–male, the other one was a male–male pair with above second-degree kinship and carried the same Y-chromosome haplogroup. This pattern might indicate that females served as key links between social classes through marriage, although larger sample sizes are needed to confirm this observation.

We identified four principal Y-chromosome haplogroups (Table S1F), each present in at least two males from the Shangshihe cemetery: (i) Q1a1a and N1a2 were prevalent in males from north–south oriented tombs, present in 11 of 12 individuals; (ii) C2b1b was present in three males—two from east–west oriented tombs (SHM019 and SSH-M85) and one from north–south oriented tomb (SSH-M4). Among them, SSH-M4 (north–south tomb) and SHM019 (east–west tomb) had close autosomal kinship with SSH-M85 from an east–west oriented tomb; (iii) O2a2b1a2a1 was found in three individuals from east–west oriented tombs, two of whom (KXHGM40 and SSHM10) were third-degree relatives.

Four of the five male–male pairs with close biological kinship on autosomes shared both tomb orientation and Y-chromosome lineages, indicating a high degree of overlap between social stratification and patrilineal boundaries, consistent with a patrilineal inheritance social structure. In contrast, mtDNA analysis revealed notably high haplotype diversity (Table S1F). Specifically, we identified 31 unique mtDNA lineages among the 38 individuals. Only 1 of the 15 relative pairs, i.e. the third-degree relatives KXHGM40 and SSHM10, shares the same mtDNA haplogroup. By integrating mtDNA and Y-chromosome analyses with autosomal kinship inference, we provide evidence supporting a predominantly patrilineal descent structure within the Shangshihe cemetery.

#### Genetic relatedness estimation for the Dongxiaoli-Bailong cemetery

The Dongxiaoli-Bailong cemetery, located in the Xiong’an New Area of Hebei Province in northern China, is a civilian cemetery dating from the late Western Han to the late Eastern Han dynasty (206 BCE–220 AD) [24]. Archaeological studies have indicated the emergence of joint burial practices for couples during the Han dynasty [23]. However, archaeological evidence alone cannot directly distinguish whether co-buried individuals were marital partners or close biological relatives (such as siblings or cousins). Moreover, due to frequent fragmentation of skeletal remains, molecular sex identification through genetic analysis is often necessary even though morphological traits (e.g. pelvis and cranium) can provide highly accurate sex assessment.

In this study, we successfully sequenced 26 genomes from the Dongxiaoli-Bailong cemetery, including 12 genomes from six double-coffin tombs: M10, M21, M26, M43, M44 and M56 (Table S1G). These ancient genomes offer an opportunity to directly investigate the biological relationships and the biological sex of these co-buried individuals. We found that these six double-coffin tombs contained one genetically female adult and one genetically male adult of comparable age at death. Moreover, they were genetically unrelated to each other, suggesting that they likely represented married couples—although no genetic evidence of offspring was detected. We also reconstructed five parent–offspring pairs, none of whom were buried together in the same tomb. This pattern further supports the observation that parent–offspring co-burial was not practiced at the Dongxiaoli-Bailong cemetery. Together, these findings may indicate that the society represented by the Han dynasty Dongxiaoli-Bailong placed greater emphasis on the relationship established by marriage alliances rather than on ancestral lineage inheritance. Notably, tomb M10 contained the co-burial of a 20 to 25-year-old adult male (DXL-M10bei) and a 6- to 8-year-old female child (DXL-M10nan) [23]. Biological kinship analyses did not identify them as a father–daughter pair, and this result is reliable because their overlapping SNPs on the 1240k panel (14 407) were sufficient to infer first- or second-degree kinship. Furthermore, archaeological evidence suggests that the scattered human bones of DXL-M10bei likely resulted from secondary relocation [24]—a practice involving the reburial of an individual who had already been buried. The joint burial of an adult male with an unrelated female child reinforces the conclusion that co-burial in this context was not necessarily based on biological kinship.

### ROH patterns for individuals from 6000 to 100 years ago in the middle Yellow River

We also performed ROH analysis on previously published genomes from 4000 years ago Yangshaocun sites [5], 3000 to 1000 years ago Lusixi sites (YR_Western_Zhou_dynasty, YR_Spring_and_Autumn_period and YR_Tang_Dynasty) [9], as well as recently published genomes from 6000 to 5000 years ago YR and Yangtze River regions [3] (Fig. S8). The ROH patterns for these published genomes have not been discussed until now. A total sum of ROH segments >20 cM indicates potential inbreeding. For Yangshao culture-related genomes (6000 to 5000-year-old), 2 of 40 individuals had long ROHs of 47 and 27 cM, respectively, which are compatible with being offspring of second or third cousins (Fig. S8A). For Longshan culture-related genomes (4500 to 4000-year-old), two of seven individuals from 4000-year-old western Henan showed long ROHs of >100 cM, indicating the existence of consanguinity, such as the mating of first cousins or closer (Fig. S8B). These results suggest the presence of low but non-negligible levels of consanguinity in Yangshao and Longshan culture-related societies in the YR region, although larger sample sizes are needed to confirm this pattern. The absence of long ROH segments in all 3000 to 100 years ago individuals from the Central Plain indicates that recent inbreeding was effectively avoided (Fig. S8C and D).

## DISCUSSION

The genetic evidence presented in this study reveals a complex pattern of population dynamics in the Central Plain of China from the Neolithic to the historical era. Three key findings reshape our understanding of prehistoric gene flow and kinship practices in this critical region.

**Figure 3.**
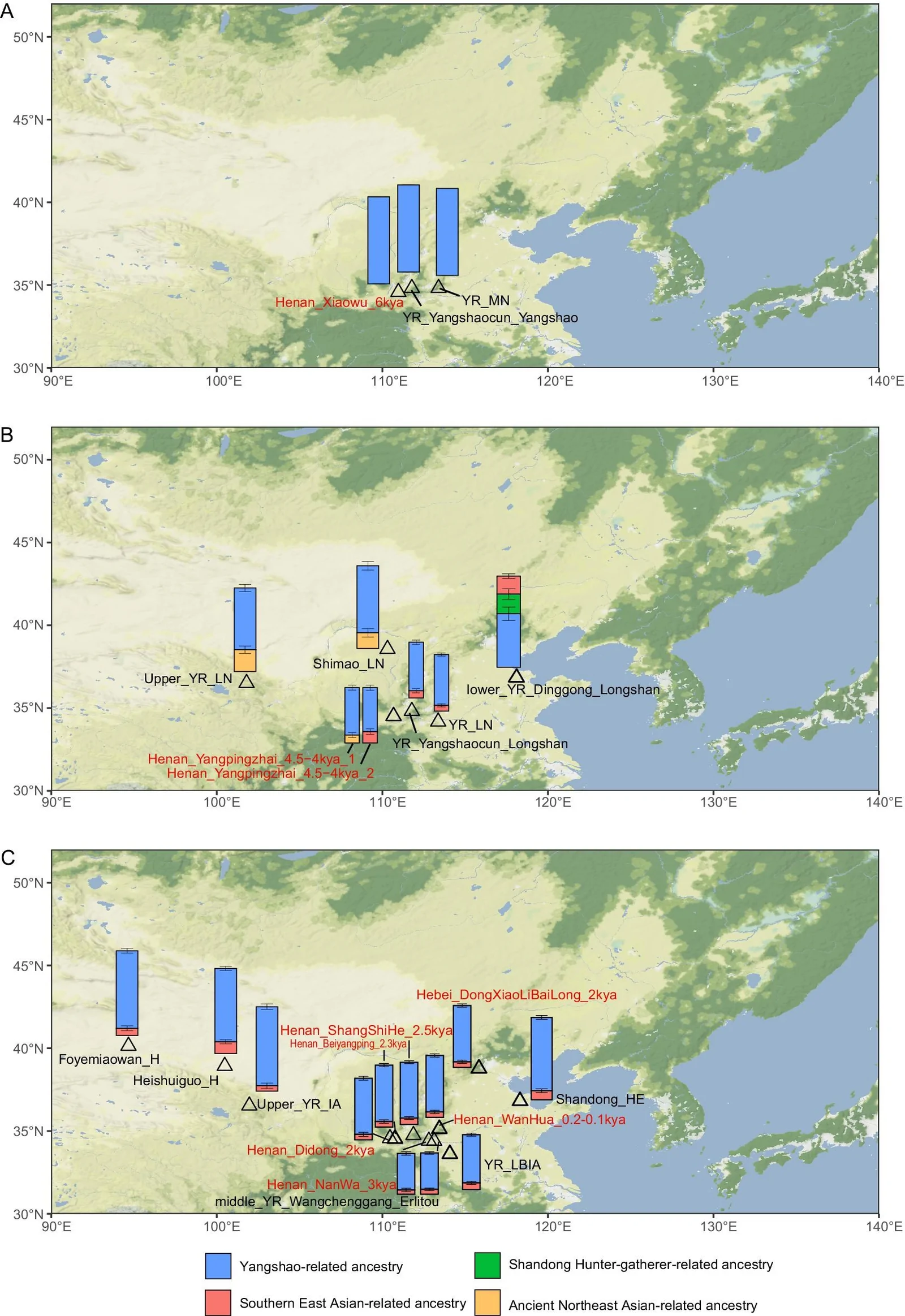
Genetic landscape in ancient YR. Well-fitted qpWave/qpAdm-based modeling results based on the autosomes for grouped-based population genetics analyses for (A) Yangshao cultural period (∼6200–5000 years ago), (B) Longshan cultural period (∼4300–4000 years ago) and (C) China’s dynastic period (∼3000–100 years ago). Error bars show one standard deviation. Raw ancestry proportions and standard error estimates are provided in Table S2D. Review drawing number: GS 京(2026)2047号.

Our newly generated Xiaowu samples represent one of the earliest Yangshao culture-related genomes in the middle YR to date. We found that early to late Yangshao culture people originated from a single ancestral source, corresponding to regional genetic continuity (Fig. 3A). The archaeological record testifies to major cultural changes in the middle YR after the Yangshao cultural period. By 4300 years ago, Neolithic cultures in western Henan appeared to be largely replaced by the Taosi culture, named after the Taosi site in southern Shanxi [7]. Recent ancient genomic research suggests that 4000-year-old individuals from the Shanxi-Shaanxi area (represented by Taosi, Shimao and Lushanmao) share an admixed profile of northern steppe and Central Plain-related ancestry [29]. An examination of genetic patterns in our newly generated 4000-year-ago individuals at the Yangpingzhai site suggests that the human populations in western Henan also display an ancestry similar to that of Shimao_LN (Fig. 3B). We did not observe a Shimao_LN-related ancestral component in people associated with the Yangshao culture in western Henan. The appearance of a Shimao_LN-related genetic component in western Henan during the Longshan period reflects a demographic connection with populations from the Shanxi-Shaanxi area. We also found that a genetic outlier at the Yangpingzhai site displayed an ancestral component that was also dominant in 4000-year-old central Henan (represented by YR_LN). This result suggests individual or small-scale population movement between central and western Henan during the Longshan cultural period.

The Shang dynasty is the first dynasty in Chinese civilization to have written records. Our newly generated ancient genomes provide direct genetic evidence that the people of the Shang dynasty in central Henan were not only unadmixed descendants of Henan Longshan culture and Erlitou culture-related people but also the direct ancestors of local 3000 to 2000 years ago people (including the Western Zhou, Eastern Zhou, Spring and Autumn and Warring States periods) and Qing dynasty people (200–100 years ago) in central Henan, suggesting long-term genetic stability in central Henan. Intriguingly, during the Longshan cultural period (4000 years ago), people from western and central Henan in the middle YR, Shandong in the lower YR and Qinghai in the upper YR were genetically heterogeneous (Fig. 3B). However, the regional differentiation between western and central Henan had disappeared by at least the Zhou dynasty, while differentiation among the middle, lower and upper YR regions had disappeared by at least the Han dynasty (Fig. 3C). These genetic shifts were all caused by the strong expansion of YR_LN-related ancestry, resulting in a highly genetically homogeneous gene pool throughout the YR. Archaeological and historical records document that the Central Plain was adjacent to several powerful polities represented by the Rong-Di and Xiongnu over the past three millennia [8]. Despite historical accounts of migrations by the Rong-Di, Xianbei and Xiongnu into the Central Plain, our genetic analysis reveals no significant corresponding demographic influx. This discordance could be attributed to several factors: the limited scale of migration such as elite-driven cultural and political integration rather than broad population admixture, or gaps in our current sampling of the relevant populations. Yet, we cannot exclude the possibility that people with non-Han identity intermarried with Han Chinese. For example, previously published ancient genomes from the Spring and Autumn period at the Lusixi site in western Henan were identified as Rong-Di according to funerary objects, but they displayed typical Central Plain-related ancestry [9]. These results support that Rong-Di who migrated into the Central Plain experienced strong genetic assimilation by local people. It is worth noting that f-statistics do not always have sufficient power to reconstruct events that involve closely related ancestries, despite increasing sample sizes. Therefore, the conclusion of genetic stability since the Shang–Qing period may be influenced by methodological limitations. The methodological limitations of our study somewhat limit our comprehensive understanding of demographic dynamics. Further research generating >0.5× shotgun genomes will be essential for haplotype-based fine-scale population substructure analysis.

We also utilized ancient genomic data to investigate social organization in Iron Age northern China through two case studies. At the Shangshihe site dating to the Spring and Autumn period, we observed a clear correlation between tomb orientation (a proxy for social status), close autosomal kinship and shared Y-chromosome lineages. Four of five closely related male–male pairs shared both tomb orientation and Y-haplogroup, strongly supporting a model of patrilineal inheritance of social status. The exceptionally high mitochondrial haplotype diversity, even among close relatives, further coincides with a patrilocal residence pattern. The pattern in which four of the five cross-orientation relative pairs involved females also suggests that women moved between kin groups or social strata through marriage, acting as genetic and social connectors in a patrilineal system. At the Dongxiaoli-Bailong site dating to the Han dynasty, we identified five parent–offspring pairs, none of whom were buried together in the same tomb. We also investigated six double-coffin tombs; each contained one biological adult male and one biological adult female of similar age. They were genetically unrelated to each other, suggesting that they likely represented married couples, although no genetic evidence of offspring was detected. These results support the archaeological hypothesis of spousal joint burial in the Han dynasty. Furthermore, we discovered a tomb in which an adult male was buried with a genetically unrelated young girl (6–8 years old). This indicates that the social rules for tomb inclusion extended beyond married couples and could incorporate individuals connected by social ties—such as adopted children—rather than biological kinship.

## Supplementary Material

nwag395_Supplemental_Files

## Data Availability

The BAM files reported in this paper have been deposited in the Genome Sequence Archive in the National Genomics Data Center, China National Center for Bioinformation/Beijing Institute of Genomics, Chinese Academy of Sciences (GSA-Human: HRA015242).
