# Supplementary material for: A 6000-year genomic transect in the middle Yellow River
Source: Natl Sci Rev. 2026 Jun 26;13(17):nwag395. doi: 10.1093/nsr/nwag395 (PMC13584218; doi:10.1093/nsr/nwag395)
Supplement: Supplementary file 1 [file nwag395_supplemental_files.zip › figureS1_mapdamage.pdf]

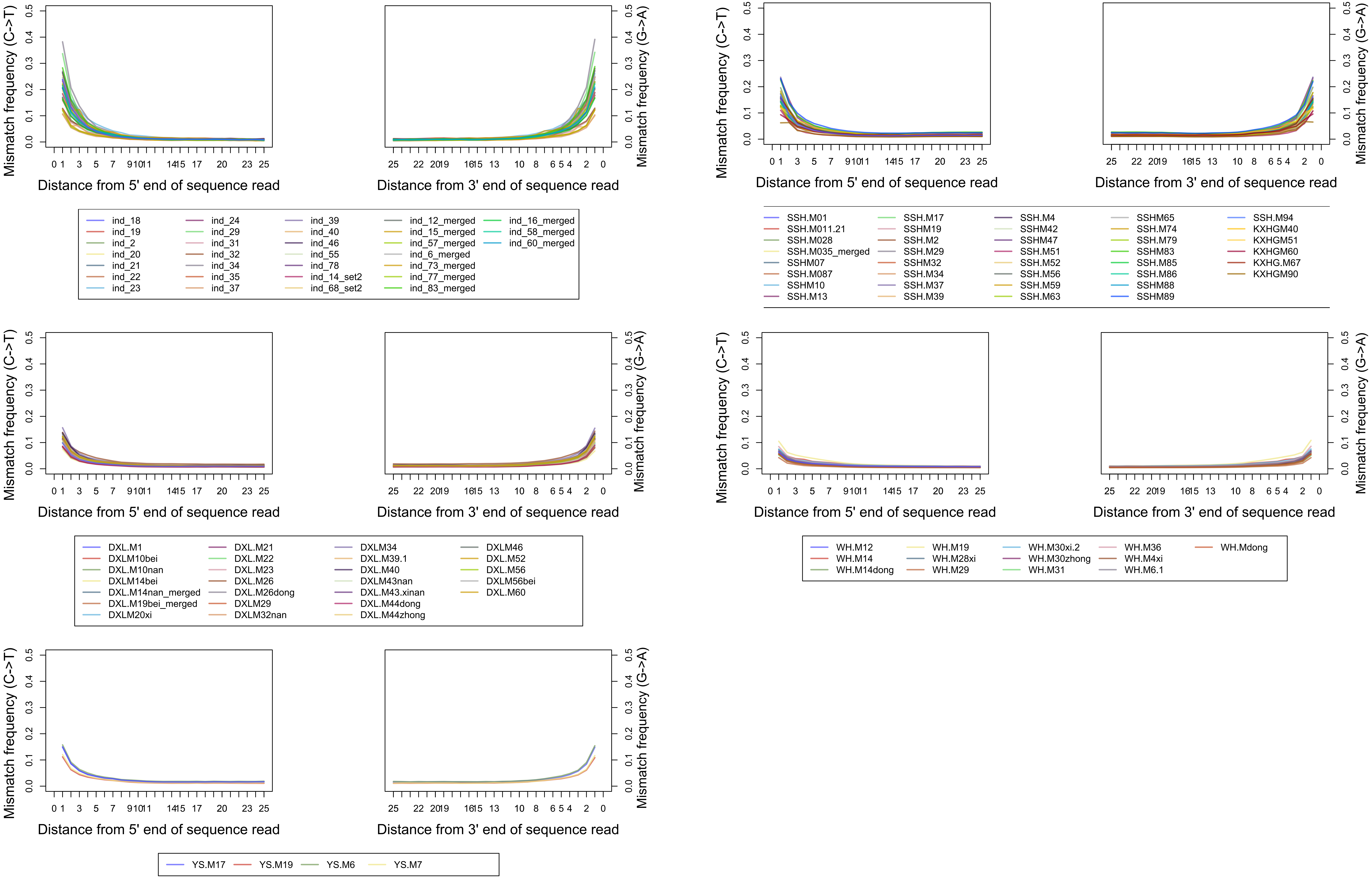
Mismatch frequency (C->T)
Mismatch frequency (G->A)
Distance from 5' end of sequence read
Distance from 3' end of sequence read
ind_18 ind_19 ind_2 ind_20 ind_21 ind_22 ind_23
ind_24 ind_29 ind_31 ind_32 ind_34 ind_35 ind_37
ind_39 ind_40 ind_46 ind_55 ind_78 ind_14_set2 ind_68_set2
ind_12_merged ind_15_merged ind_57_merged ind_6_merged ind_73_merged ind_77_merged ind_83_merged
ind_16_merged ind_58_merged ind_60_merged
SSH.M01 SSH.M011.21 SSH.M028 SSH.M035_merged SSHM07 SSH.M087 SSHM10 SSH.M13
SSH.M17 SSHM19 SSH.M2 SSH.M29 SSHM32 SSH.M34 SSH.M37 SSH.M39
SSH.M4 SSHM42 SSHM47 SSH.M51 SSH.M52 SSH.M56 SSH.M59 SSH.M63
SSHM65 SSH.M74 SSH.M79 SSHM83 SSH.M85 SSH.M86 SSHM88 SSHM89
SSH.M94 KXHGM40 KXHGM51 KXHGM60 KXHG.M67 KXHGM90
DXL.M1 DXLM10bei DXL.M10nan DXLM14bei DXL.M14nan_merged DXL.M19bei_merged DXLM20xi
DXL.M21 DXL.M22 DXL.M23 DXL.M26 DXL.M26dong DXLM29 DXLM32nan
DXLM34 DXL.M39.1 DXL.M40 DXLM43nan DXL.M43.xinan DXL.M44dong DXL.M44zhong
DXLM46 DXL.M52 DXL.M56 DXLM56bei DXL.M60
WH.M12 WH.M14 WH.M14dong
WH.M19 WH.M28xi WH.M29
WH.M30xi.2 WH.M30zhong WH.M31
WH.M36 WH.M4xi WH.M6.1
WH.Mdong
YS.M17 YS.M19 YS.M6 YS.M7
