# Supplementary material for: A 6000-year genomic transect in the middle Yellow River
Source: Natl Sci Rev. 2026 Jun 26;13(17):nwag395. doi: 10.1093/nsr/nwag395 (PMC13584218; doi:10.1093/nsr/nwag395)
Supplement: Supplementary file 1 [file nwag395_supplemental_files.zip › figureS2_outgroup_f3_errbar.pdf]

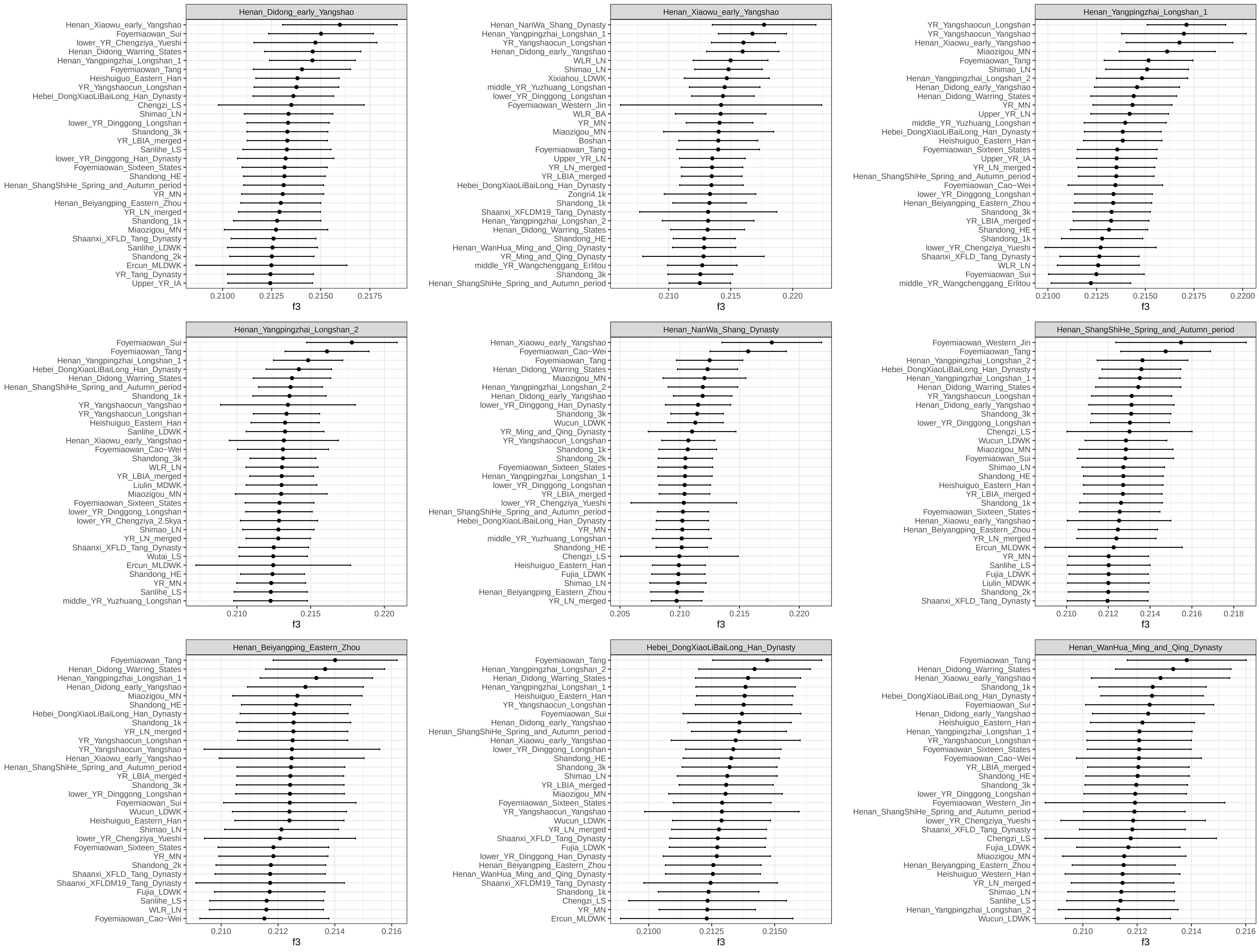
Henan_Didong_early_Yangshao
Henan_Xiaowu_early_Yangshao
Foyemiaowan_Sui
lower_YR_Chengziya_Yueshi
Henan_Didong_Warring_States
Henan_Yangpingzhai_Longshan_1
Foyemiaowan_Tang
Heishuiguo_Eastern_Han
YR_Yangshaocun_Longshan
Hebei_DongXiaoLiBaiLong_Han_Dynasty
Chengzi_LS
Shimao_LN
lower_YR_Dinggong_Longshan
Shandong_3k
YR_LBIA_merged
Sanlihe_LS
lower_YR_Dinggong_Han_Dynasty
Foyemiaowan_Sixteen_States
Shandong_HE
Henan_ShangShiHe_Spring_and_Autumn_period
YR_MN
Henan_Beiyangping_Eastern_Zhou
YR_LN_merged
Shandong_1k
Miaozigou_MN
Shaanxi_XFLD_Tang_Dynasty
Sanlihe_LDWK
Shandong_2k
Ercun_MLDWK
YR_Tang_Dynasty
Upper_YR_IA
0.2100
0.2125
0.2150
0.2175
f3
Henan_Xiaowu_early_Yangshao
Henan_NanWa_Shang_Dynasty
Henan_Yangpingzhai_Longshan_1
YR_Yangshaocun_Longshan
Henan_Didong_early_Yangshao
WLR_LN
Shimao_LN
Xixiahou_LDWK
middle_YR_Yuzhuang_Longshan
lower_YR_Dinggong_Longshan
Foyemiaowan_Western_Jin
WLR_BA
YR_MN
Miaozigou_MN
Boshan
Foyemiaowan_Tang
Upper_YR_LN
YR_LN_merged
YR_LBIA_merged
Hebei_DongXiaoLiBaiLong_Han_Dynasty
Zongri4.1k
Shandong_1k
Shaanxi_XFLDM19_Tang_Dynasty
Henan_Yangpingzhai_Longshan_2
Henan_Didong_Warring_States
Shandong_HE
Henan_WanHua_Ming_and_Qing_Dynasty
YR_Ming_and_Qing_Dynasty
middle_YR_Wangchenggang_Erlitou
Shandong_3k
Henan_ShangShiHe_Spring_and_Autumn_period
0.210
0.215
0.220
f3
Henan_Yangpingzhai_Longshan_1
YR_Yangshaocun_Longshan
YR_Yangshaocun_Yangshao
Henan_Xiaowu_early_Yangshao
Miaozigou_MN
Foyemiaowan_Tang
Shimao_LN
Henan_Yangpingzhai_Longshan_2
Henan_Didong_early_Yangshao
Henan_Didong_Warring_States
YR_MN
Upper_YR_LN
middle_YR_Yuzhuang_Longshan
Hebei_DongXiaoLiBaiLong_Han_Dynasty
Heishuiguo_Eastern_Han
Foyemiaowan_Sixteen_States
Upper_YR_IA
YR_LN_merged
Henan_ShangShiHe_Spring_and_Autumn_period
Foyemiaowan_Cao−Wei
lower_YR_Dinggong_Longshan
Henan_Beiyangping_Eastern_Zhou
Shandong_3k
YR_LBIA_merged
Shandong_HE
Shandong_1k
lower_YR_Chengziya_Yueshi
Shaanxi_XFLD_Tang_Dynasty
WLR_LN
Foyemiaowan_Sui
middle_YR_Wangchenggang_Erlitou
0.2100
0.2125
0.2150
0.2175
0.2200
f3
Henan_Yangpingzhai_Longshan_2
Foyemiaowan_Sui
Foyemiaowan_Tang
Henan_Yangpingzhai_Longshan_1
Hebei_DongXiaoLiBaiLong_Han_Dynasty
Henan_Didong_Warring_States
Henan_ShangShiHe_Spring_and_Autumn_period
Shandong_1k
YR_Yangshaocun_Yangshao
YR_Yangshaocun_Longshan
Heishuiguo_Eastern_Han
Sanlihe_LDWK
Henan_Xiaowu_early_Yangshao
Foyemiaowan_Cao−Wei
Shandong_3k
WLR_LN
YR_LBIA_merged
Liulin_MDWK
Miaozigou_MN
Foyemiaowan_Sixteen_States
lower_YR_Dinggong_Longshan
lower_YR_Chengziya_2.5kya
Shimao_LN
YR_LN_merged
Shaanxi_XFLD_Tang_Dynasty
Wutai_LS
Ercun_MLDWK
Shandong_HE
YR_MN
Sanlihe_LS
middle_YR_Yuzhuang_Longshan
0.210
0.215
0.220
f3
Henan_NanWa_Shang_Dynasty
Henan_Xiaowu_early_Yangshao
Foyemiaowan_Cao−Wei
Foyemiaowan_Tang
Henan_Didong_Warring_States
Miaozigou_MN
Henan_Yangpingzhai_Longshan_2
Henan_Didong_early_Yangshao
lower_YR_Dinggong_Han_Dynasty
Shandong_3k
Wucun_LDWK
YR_Ming_and_Qing_Dynasty
YR_Yangshaocun_Longshan
Shandong_1k
Shandong_2k
Foyemiaowan_Sixteen_States
Henan_Yangpingzhai_Longshan_1
lower_YR_Dinggong_Longshan
YR_LBIA_merged
lower_YR_Chengziya_Yueshi
Henan_ShangShiHe_Spring_and_Autumn_period
Hebei_DongXiaoLiBaiLong_Han_Dynasty
YR_MN
middle_YR_Yuzhuang_Longshan
Shandong_HE
Chengzi_LS
Heishuiguo_Eastern_Han
Fujia_LDWK
Shimao_LN
Henan_Beiyangping_Eastern_Zhou
YR_LN_merged
0.205
0.210
0.215
0.220
f3
Henan_ShangShiHe_Spring_and_Autumn_period
Foyemiaowan_Western_Jin
Foyemiaowan_Tang
Henan_Yangpingzhai_Longshan_2
Hebei_DongXiaoLiBaiLong_Han_Dynasty
Henan_Yangpingzhai_Longshan_1
Henan_Didong_Warring_States
YR_Yangshaocun_Longshan
Henan_Didong_early_Yangshao
Shandong_3k
lower_YR_Dinggong_Longshan
Chengzi_LS
Wucun_LDWK
Miaozigou_MN
Foyemiaowan_Sui
Shimao_LN
Shandong_HE
Heishuiguo_Eastern_Han
YR_LBIA_merged
Shandong_1k
Foyemiaowan_Sixteen_States
Henan_Xiaowu_early_Yangshao
Henan_Beiyangping_Eastern_Zhou
YR_LN_merged
Ercun_MLDWK
YR_MN
Sanlihe_LS
Fujia_LDWK
Liulin_MDWK
Shandong_2k
Shaanxi_XFLD_Tang_Dynasty
0.210
0.212
0.214
0.216
0.218
f3
Henan_Beiyangping_Eastern_Zhou
Foyemiaowan_Tang
Henan_Didong_Warring_States
Henan_Yangpingzhai_Longshan_1
Henan_Didong_early_Yangshao
Miaozigou_MN
Shandong_HE
Hebei_DongXiaoLiBaiLong_Han_Dynasty
Shandong_1k
YR_LN_merged
YR_Yangshaocun_Longshan
YR_Yangshaocun_Yangshao
Henan_Xiaowu_early_Yangshao
Henan_ShangShiHe_Spring_and_Autumn_period
YR_LBIA_merged
Shandong_3k
lower_YR_Dinggong_Longshan
Foyemiaowan_Sui
Wucun_LDWK
Heishuiguo_Eastern_Han
Shimao_LN
lower_YR_Chengziya_Yueshi
Foyemiaowan_Sixteen_States
YR_MN
Shandong_2k
Shaanxi_XFLD_Tang_Dynasty
Shaanxi_XFLDM19_Tang_Dynasty
Fujia_LDWK
Sanlihe_LS
WLR_LN
Foyemiaowan_Cao−Wei
0.210
0.212
0.214
0.216
f3
Hebei_DongXiaoLiBaiLong_Han_Dynasty
Foyemiaowan_Tang
Henan_Yangpingzhai_Longshan_2
Henan_Didong_Warring_States
Henan_Yangpingzhai_Longshan_1
Heishuiguo_Eastern_Han
YR_Yangshaocun_Longshan
Foyemiaowan_Sui
Henan_Didong_early_Yangshao
Henan_ShangShiHe_Spring_and_Autumn_period
Henan_Xiaowu_early_Yangshao
lower_YR_Dinggong_Longshan
Shandong_HE
Shandong_3k
Shimao_LN
YR_LBIA_merged
Miaozigou_MN
Foyemiaowan_Sixteen_States
YR_Yangshaocun_Yangshao
Wucun_LDWK
YR_LN_merged
Shaanxi_XFLD_Tang_Dynasty
Fujia_LDWK
lower_YR_Dinggong_Han_Dynasty
Henan_Beiyangping_Eastern_Zhou
Henan_WanHua_Ming_and_Qing_Dynasty
Shaanxi_XFLDM19_Tang_Dynasty
Shandong_1k
Chengzi_LS
YR_MN
Ercun_MLDWK
0.2100
0.2125
0.2150
f3
Henan_WanHua_Ming_and_Qing_Dynasty
Foyemiaowan_Tang
Henan_Didong_Warring_States
Henan_Xiaowu_early_Yangshao
Shandong_1k
Hebei_DongXiaoLiBaiLong_Han_Dynasty
Foyemiaowan_Sui
Henan_Didong_early_Yangshao
Heishuiguo_Eastern_Han
Henan_Yangpingzhai_Longshan_1
YR_Yangshaocun_Longshan
Foyemiaowan_Sixteen_States
Foyemiaowan_Cao−Wei
YR_LBIA_merged
Shandong_HE
Shandong_3k
lower_YR_Dinggong_Longshan
Foyemiaowan_Western_Jin
Henan_ShangShiHe_Spring_and_Autumn_period
lower_YR_Chengziya_Yueshi
Shaanxi_XFLD_Tang_Dynasty
Chengzi_LS
Fujia_LDWK
Miaozigou_MN
Henan_Beiyangping_Eastern_Zhou
Heishuiguo_Western_Han
YR_LN_merged
Shimao_LN
Sanlihe_LS
Henan_Yangpingzhai_Longshan_2
Wucun_LDWK
0.210
0.212
0.214
0.216
f3
