# Supplementary material for: A 6000-year genomic transect in the middle Yellow River
Source: Natl Sci Rev. 2026 Jun 26;13(17):nwag395. doi: 10.1093/nsr/nwag395 (PMC13584218; doi:10.1093/nsr/nwag395)
Supplement: Supplementary file 1 [file nwag395_supplemental_files.zip › figureS4_f4_Henan_Yangpingzhai_4.5-4kya_1.pdf]

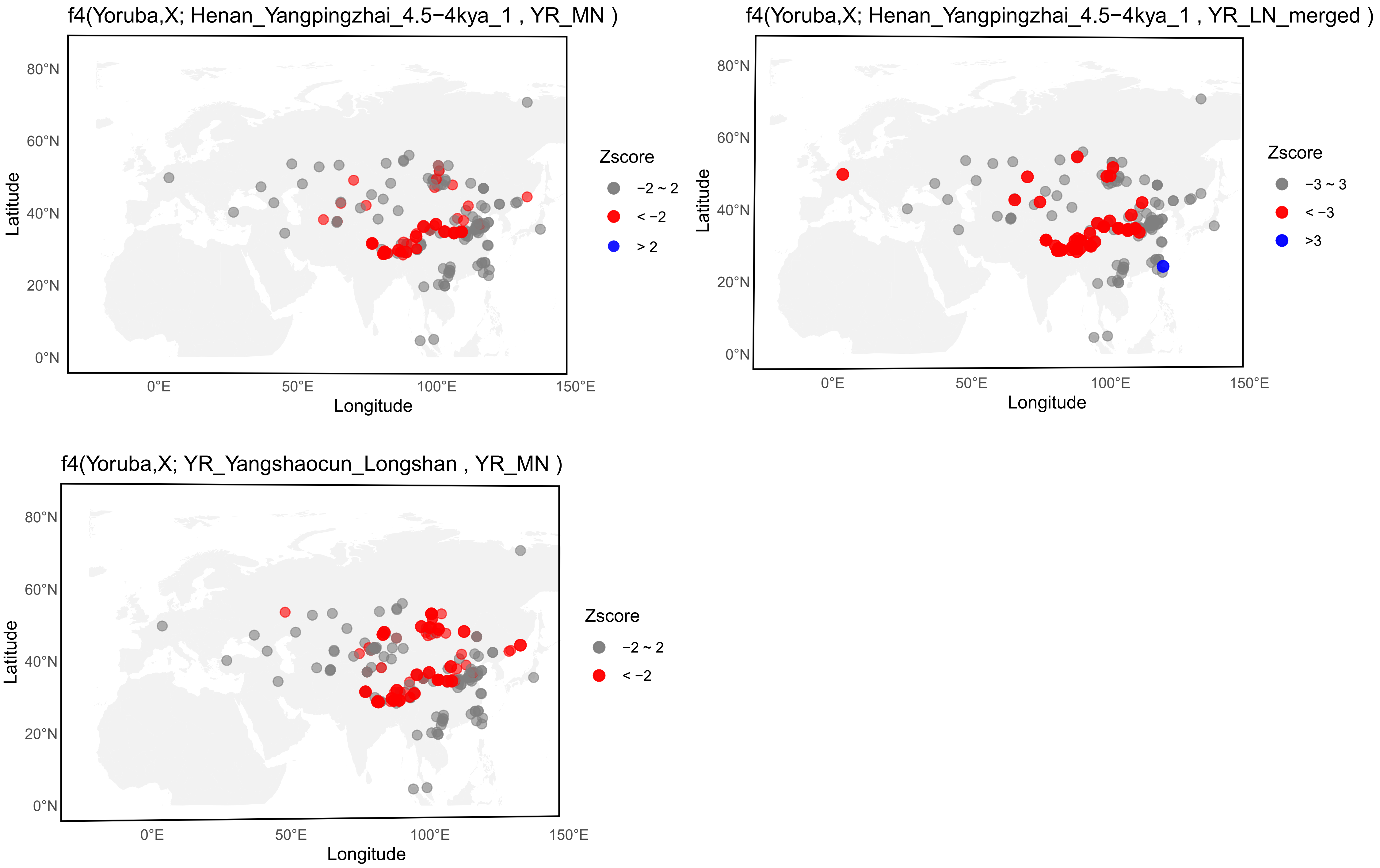
f4(Yoruba,X; Henan_Yangpingzhai_4.5−4kya_1 , YR_MN)
80°N
60°N
40°N
20°N
0°N
Latitude
0°E
50°E
100°E
150°E
Longitude
Zscore
−2 ~ 2
< −2
> 2
f4(Yoruba,X; Henan_Yangpingzhai_4.5−4kya_1 , YR_LN_merged)
80°N
60°N
40°N
20°N
0°N
Latitude
0°E
50°E
100°E
150°E
Longitude
Zscore
−3 ~ 3
< −3
>3
f4(Yoruba,X; YR_Yangshaocun_Longshan , YR_MN)
80°N
60°N
40°N
20°N
0°N
Latitude
0°E
50°E
100°E
150°E
Longitude
Zscore
−2 ~ 2
< −2
