# Supplementary material for: A 6000-year genomic transect in the middle Yellow River
Source: Natl Sci Rev. 2026 Jun 26;13(17):nwag395. doi: 10.1093/nsr/nwag395 (PMC13584218; doi:10.1093/nsr/nwag395)
Supplement: Supplementary file 1 [file nwag395_supplemental_files.zip › figureS5_f4_Henan_Yangpingzhai_4.5-4kya_2.pdf]

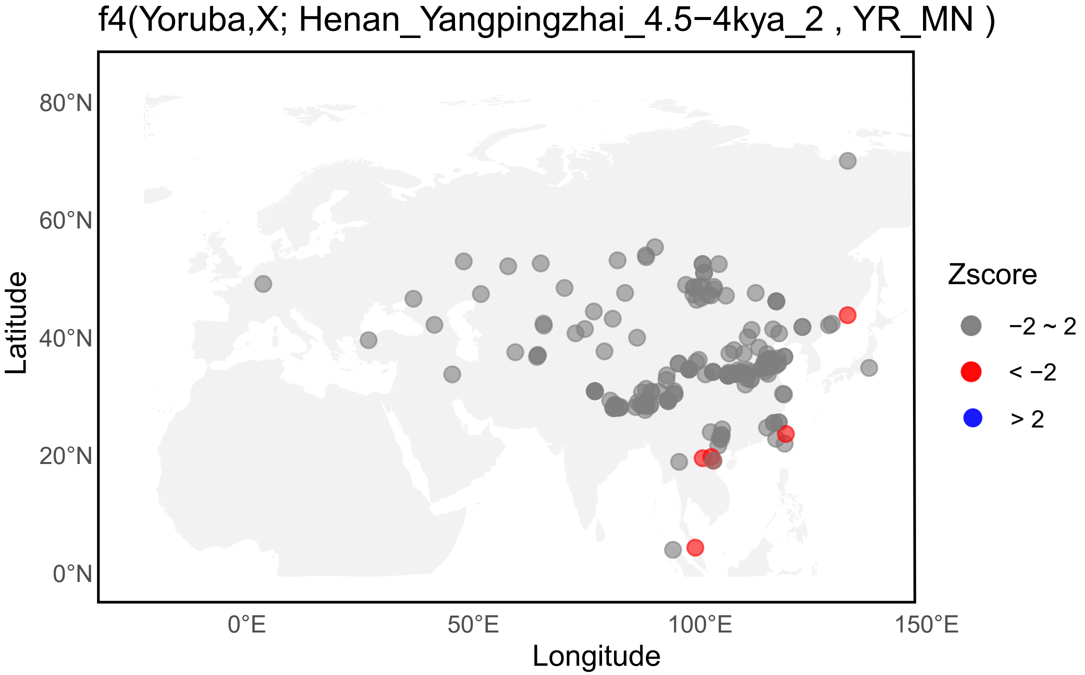

f4(Yoruba,X; Henan_Yangpingzhai_4.5−4kya_2 , YR_MN)
80°N
60°N
40°N
20°N
0°N
Latitude
0°E
50°E
100°E
150°E
Longitude
Zscore
−2 ~ 2
< −2
> 2
