# Supplementary material for: A 6000-year genomic transect in the middle Yellow River
Source: Natl Sci Rev. 2026 Jun 26;13(17):nwag395. doi: 10.1093/nsr/nwag395 (PMC13584218; doi:10.1093/nsr/nwag395)
Supplement: Supplementary file 1 [file nwag395_supplemental_files.zip › figureS6_f4_YR_LN_YR_LBIA_Erlitou.pdf]

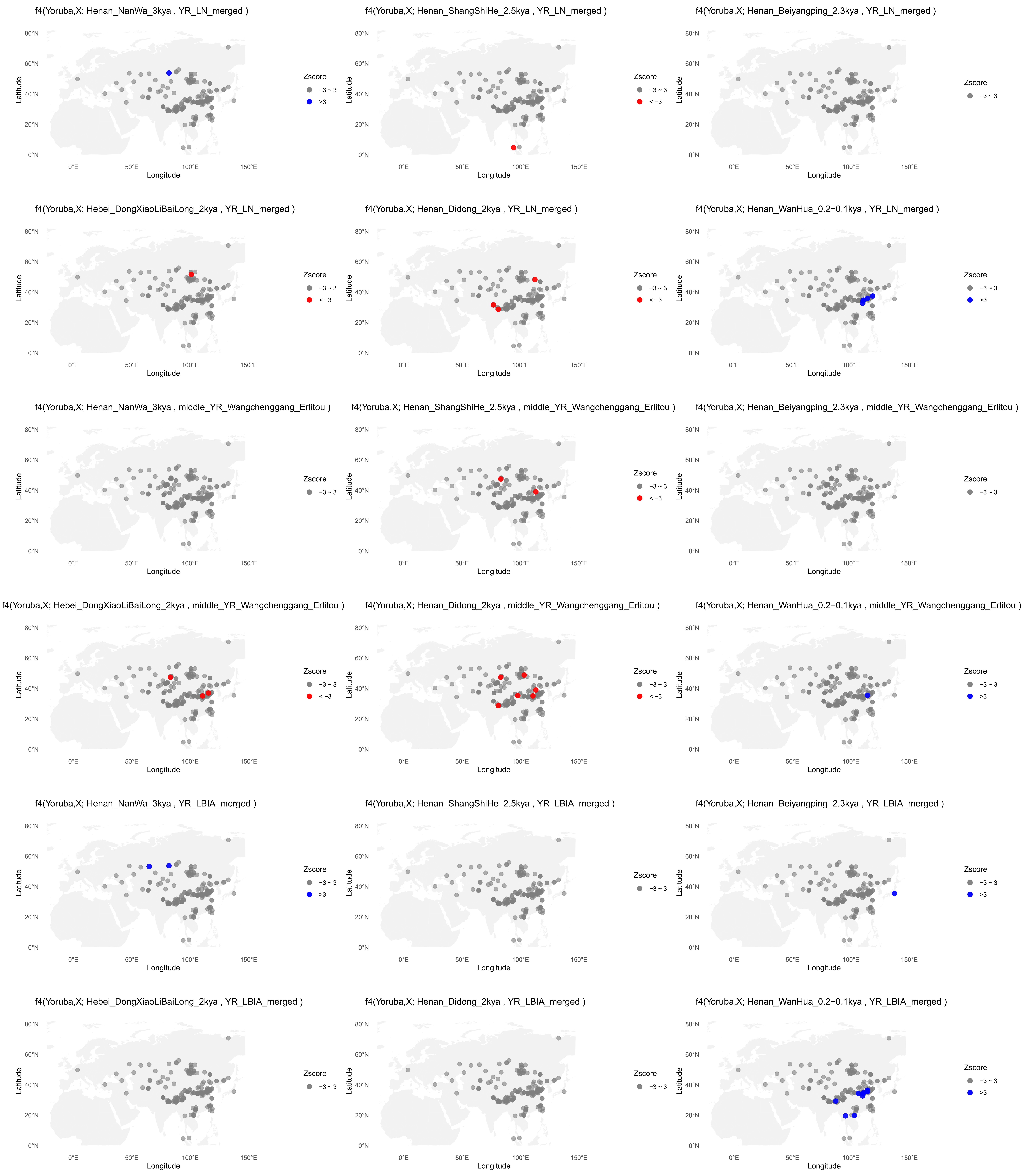

f4(Yoruba,X; Henan_NanWa_3kya , YR_LN_merged)
Zscore
−3 ~ 3
>3
f4(Yoruba,X; Henan_ShangShiHe_2.5kya , YR_LN_merged)
Zscore
−3 ~ 3
< −3
f4(Yoruba,X; Henan_Beiyangping_2.3kya , YR_LN_merged)
Zscore
−3 ~ 3
f4(Yoruba,X; Hebei_DongXiaoLiBaiLong_2kya , YR_LN_merged)
Zscore
−3 ~ 3
< −3
f4(Yoruba,X; Henan_Didong_2kya , YR_LN_merged)
Zscore
−3 ~ 3
< −3
f4(Yoruba,X; Henan_WanHua_0.2−0.1kya , YR_LN_merged)
Zscore
−3 ~ 3
>3
f4(Yoruba,X; Henan_NanWa_3kya , middle_YR_Wangchenggang_Erlitou)
Zscore
−3 ~ 3
f4(Yoruba,X; Henan_ShangShiHe_2.5kya , middle_YR_Wangchenggang_Erlitou)
Zscore
−3 ~ 3
< −3
f4(Yoruba,X; Henan_Beiyangping_2.3kya , middle_YR_Wangchenggang_Erlitou)
Zscore
−3 ~ 3
f4(Yoruba,X; Hebei_DongXiaoLiBaiLong_2kya , middle_YR_Wangchenggang_Erlitou)
Zscore
−3 ~ 3
< −3
f4(Yoruba,X; Henan_Didong_2kya , middle_YR_Wangchenggang_Erlitou)
Zscore
−3 ~ 3
< −3
f4(Yoruba,X; Henan_WanHua_0.2−0.1kya , middle_YR_Wangchenggang_Erlitou)
Zscore
−3 ~ 3
>3
f4(Yoruba,X; Henan_NanWa_3kya , YR_LBIA_merged)
Zscore
−3 ~ 3
>3
f4(Yoruba,X; Henan_ShangShiHe_2.5kya , YR_LBIA_merged)
Zscore
−3 ~ 3
f4(Yoruba,X; Henan_Beiyangping_2.3kya , YR_LBIA_merged)
Zscore
−3 ~ 3
>3
f4(Yoruba,X; Hebei_DongXiaoLiBaiLong_2kya , YR_LBIA_merged)
Zscore
−3 ~ 3
f4(Yoruba,X; Henan_Didong_2kya , YR_LBIA_merged)
Zscore
−3 ~ 3
f4(Yoruba,X; Henan_WanHua_0.2−0.1kya , YR_LBIA_merged)
Zscore
−3 ~ 3
>3
Latitude
Longitude
0°N
20°N
40°N
60°N
80°N
0°E
50°E
100°E
150°E
