# Supplementary material for: A 6000-year genomic transect in the middle Yellow River
Source: Natl Sci Rev. 2026 Jun 26;13(17):nwag395. doi: 10.1093/nsr/nwag395 (PMC13584218; doi:10.1093/nsr/nwag395)
Supplement: Supplementary file 1 [file nwag395_supplemental_files.zip › figureS7_f4_Lingbao_4_3kya.pdf]

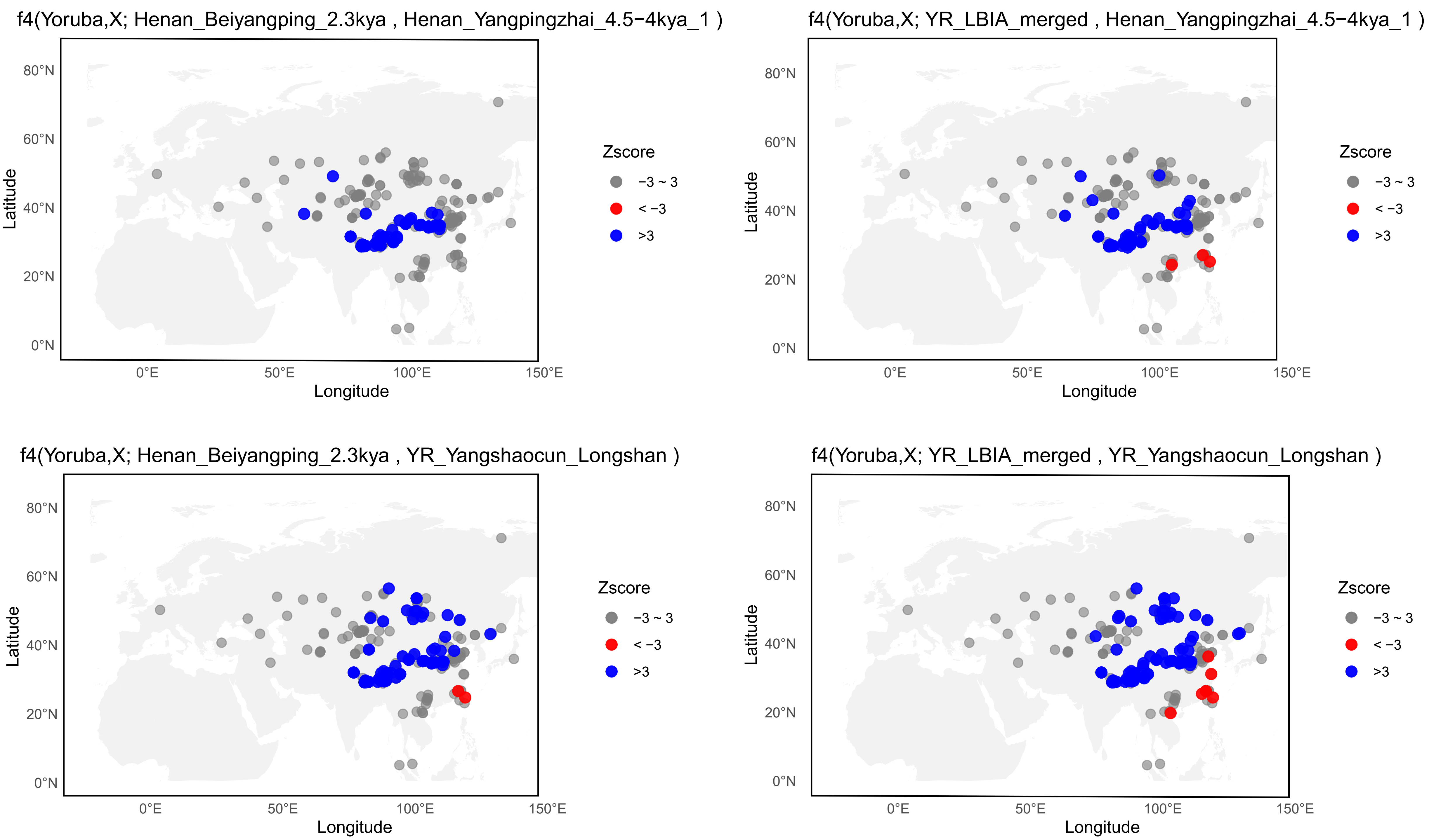

f4(Yoruba,X; Henan_Beiyangping_2.3kya , Henan_Yangpingzhai_4.5−4kya_1)
f4(Yoruba,X; YR_LBIA_merged , Henan_Yangpingzhai_4.5−4kya_1)
f4(Yoruba,X; Henan_Beiyangping_2.3kya , YR_Yangshaocun_Longshan)
f4(Yoruba,X; YR_LBIA_merged , YR_Yangshaocun_Longshan)
Latitude
80°N
60°N
40°N
20°N
0°N
Longitude
0°E
50°E
100°E
150°E
Zscore
−3 ~ 3
< −3
>3
