# Supplementary material for: A 6000-year genomic transect in the middle Yellow River
Source: Natl Sci Rev. 2026 Jun 26;13(17):nwag395. doi: 10.1093/nsr/nwag395 (PMC13584218; doi:10.1093/nsr/nwag395)
Supplement: Supplementary file 1 [file nwag395_supplemental_files.zip › figureS8_ROH_bars.pdf]

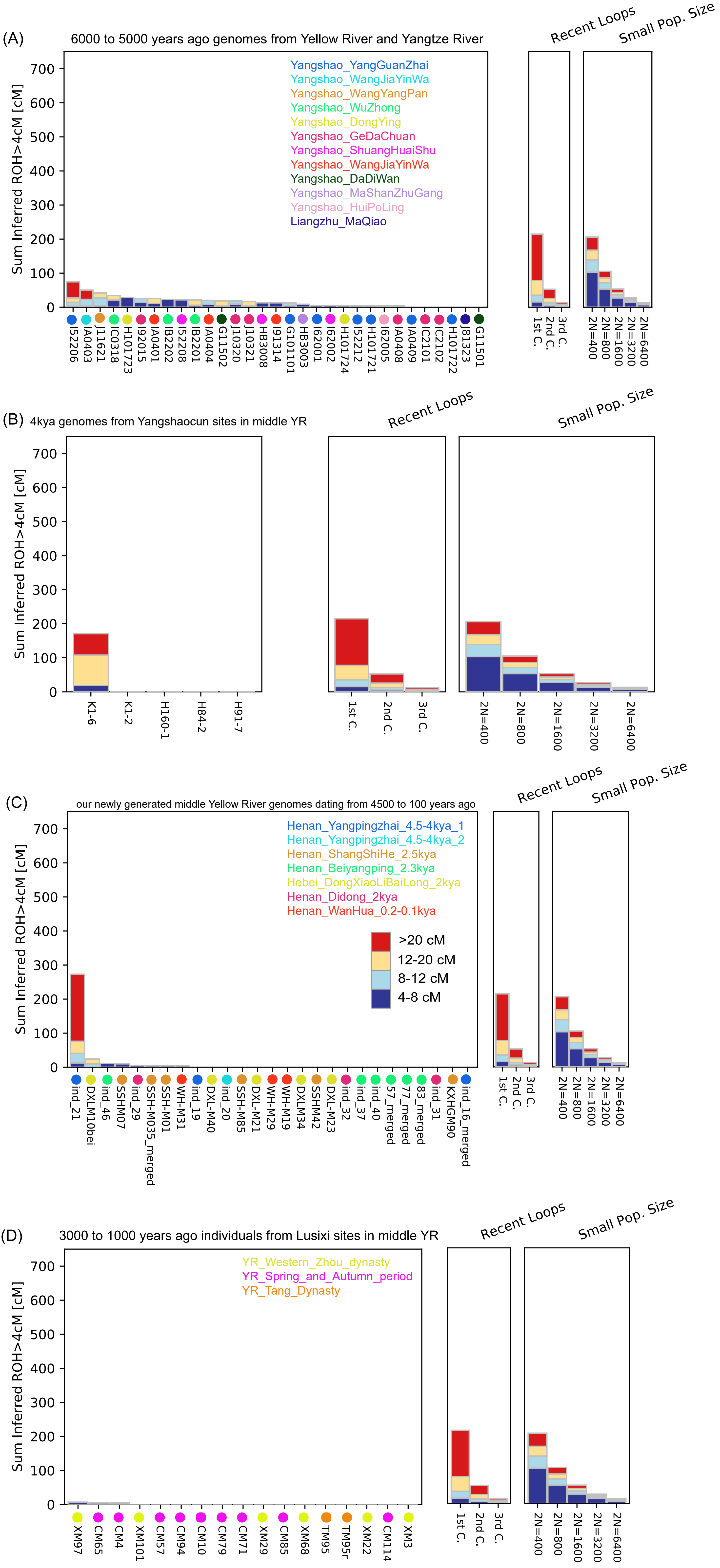

(A)
6000 to 5000 years ago genomes from Yellow River and Yangtze River
Recent Loops
Small Pop. Size
Sum Inferred ROH>4cM [cM]
Yangshao_YangGuanZhai
Yangshao_WangJiaYinWa
Yangshao_WangYangPan
Yangshao_WuZhong
Yangshao_DongYing
Yangshao_GeDaChuan
Yangshao_ShuangHuaiShu
Yangshao_WangJiaYinWa
Yangshao_DaDiWan
Yangshao_MaShanZhuGang
Yangshao_HuiPoLing
Liangzhu_MaQiao
I52206 IA0403 J11621 IC0318 H101723 I92015 IA0401 IB2202 I52208 IB2201 IA0404 G11502 J10320 J10321 HB3008 I91314 G101101 HB3003 I62001 I62002 H101724 I52212 H101721 I62005 IA0408 IA0409 IC2101 IC2102 H101722 J81323 G11501
1st C. 2nd C. 3rd C.
2N=400 2N=800 2N=1600 2N=3200 2N=6400
(B)
4kya genomes from Yangshaocun sites in middle YR
Recent Loops
Small Pop. Size
Sum Inferred ROH>4cM [cM]
K1-6 K1-2 H160-1 H84-2 H91-7
1st C. 2nd C. 3rd C.
2N=400 2N=800 2N=1600 2N=3200 2N=6400
(C)
our newly generated middle Yellow River genomes dating from 4500 to 100 years ago
Recent Loops
Small Pop. Size
Sum Inferred ROH>4cM [cM]
Henan_Yangpingzhai_4.5-4kya_1
Henan_Yangpingzhai_4.5-4kya_2
Henan_ShangShiHe_2.5kya
Henan_Beiyangping_2.3kya
Hebei_DongXiaoLiBaiLong_2kya
Henan_Didong_2kya
Henan_WanHua_0.2-0.1kya
>20 cM
12-20 cM
8-12 cM
4-8 cM
ind_21 DXLM10bei ind_46 SSHM07 ind_29 SSH-M035_merged SSH-M01 WH-M31 ind_19 DXL-M40 ind_20 SSH-M85 DXL-M21 WH-M29 WH-M19 DXLM34 SSHM42 DXL-M23 ind_32 ind_37 ind_40 57_merged 77_merged 83_merged ind_31 KXHGM90 ind_16_merged
1st C. 2nd C. 3rd C.
2N=400 2N=800 2N=1600 2N=3200 2N=6400
(D)
3000 to 1000 years ago individuals from Lusixi sites in middle YR
Recent Loops
Small Pop. Size
Sum Inferred ROH>4cM [cM]
YR_Western_Zhou_dynasty
YR_Spring_and_Autumn_period
YR_Tang_Dynasty
XM97 CM65 CM4 XM101 CM57 CM94 CM10 CM79 CM71 XM29 CM85 XM68 TM95 TM95r XM22 CM114 XM3
1st C. 2nd C. 3rd C.
2N=400 2N=800 2N=1600 2N=3200 2N=6400
0 100 200 300 400 500 600 700
