# Supplementary material for: A 6000-year genomic transect in the middle Yellow River
Source: Natl Sci Rev. 2026 Jun 26;13(17):nwag395. doi: 10.1093/nsr/nwag395 (PMC13584218; doi:10.1093/nsr/nwag395)
Supplement: Supplementary file 1 [file nwag395_supplemental_files.zip › Supplementary figure legends.docx]

**Supplemental figure titles and legends**

**Figure S1.** Ancient DNA damage pattern for newly generated individuals. The plot of terminal damage pattern for double-stranded libraries without UDG treatment for our newly generated individuals from middle YR. All individuals displayed the typical damage pattern at both ends (5’ C>T and 3’ G>A).

**Figure S2.** Outgroup f3 statistics in the form of f3 (target, Reference; Yoruba) based on 1240k dataset is used to calculate the shared genetic drift between target and Reference after divergence from the outgroup Yoruba. See also Table S2A.

**Figure S3.** F4 statistics in the form of f4(Yoruba, X; Henan_Xiaowu_6kya, YR_MN). The geographic locations of the ancient Eurasian populations (the "X" in the f4 test) are shown as dots, with colors scaled according to the Z-scores. Specifically, dots are colored blue for positive significant values (Z-scores > 2), red for negative significant values (Z-scores < –2), and gray for non-significant values (-2 <Z-scores< 2). See also Table S2C.

**Figure S4.** F4 statistics in the form of f4(Yoruba, X; Henan_Yangpingzhai_4.5-4kya_1, YR_MN/YR_LN_merged) and f4(Yoruba, X; YR_Yangshaocun_Longshan, YR_MN). The geographic locations of the ancient Eurasian populations (the "X" in the f4 test) are shown as dots, with colors scaled according to the Z-scores. Specifically, dots are colored blue for positive significant values (Z-scores > 2), red for negative significant values (Z-scores < –2), and gray for non-significant values (-2<Z-scores<2). See also Table S2C.

**Figure S5.** F4 statistics in the form of f4(Yoruba, X; Henan_Yangpingzhai_4.5-4kya_2, YR_MN). The geographic locations of the ancient Eurasian populations (the "X" in the f4 test) are shown as dots, with colors scaled according to the Z-scores. Specifically, dots are colored blue for positive significant values (Z-scores > 2), red for negative significant values (Z-scores < –2), and gray for non-significant values (-2<Z-scores<2). See also Table S2C.

**Figure S6.** F4 statistics in the form of f4(Yoruba, X; newly generated 3000 to 100 years ago ancient middle YR groups, YR_LN_merged/YR_LBIA_merged/middle_YR_Wangchenggang_Erlitou). The geographic locations of the ancient Eurasian populations (the "X" in the f4 test) are shown as dots, with colors scaled according to the Z-scores. Specifically, dots are colored blue for positive significant values (Z-scores > 3), red for negative significant values (Z-scores < –3), and gray for non-significant values (-3<Z-scores<3). See also Table S2C.

**Figure S7.** F4 statistics in the form of f4(Yoruba, X; Henan_Beiyangping_2.3kya/YR_LBIA_merged, YR_Yangshaocun_Longshan/Henan_Yangpingzhai_4.5-4kya_1). The geographic locations of the ancient Eurasian populations (the "X" in the f4 test) are shown as dots, with colors scaled according to the Z-scores. Specifically, dots are colored blue for positive significant values (Z-scores > 3), red for negative significant values (Z-scores < –3), and gray for non-significant values (-3<Z-scores<3). See also Table S2C.

**Figure S8.** ROH analysis. The ROH patterns for (A) recently published 6000-5000 years ago individuals from Yellow River and Yangtze River; (B) previously published 4000 years ago individuals from Yangshaocun sites; (C) our newly generated 4500 to 100 years ago individuals from middle Yellow River and (D) previously published 3000 to 1000 years ago individuals from Lusixi sites. Individuals possessed more than 400,000 SNPs on the 1240k panel were included. Observed and expected ROH blocks above 4 cM were plotted using hapROH.
