# Supplementary material for: A 6000-year genomic transect in the middle Yellow River
Source: Natl Sci Rev. 2026 Jun 26;13(17):nwag395. doi: 10.1093/nsr/nwag395 (PMC13584218; doi:10.1093/nsr/nwag395)
Supplement: Supplementary file 1 [file nwag395_supplemental_files.zip › Supplementary methods.docx]

**Methods and Materials**

**Archaeological background of studied sites**

We collected 119 ancient samples from 8 sites in the Middle Yellow River (Figure 1 and Table S1A). Approval for their use was curated by co-authors and obtained with permission from the respective provincial archaeology institutes or universities that managed the samples. Permission and oversight were also provided by the institutional review board of the Ethics Committee for Biological Research at Xiamen University. Our newly generated ancient genomes cover a period ranging from the Neolithic to historical time, including early Yangshao (n=8), Longshan (n=9), Shang (n=4), Eastern Zhou (n=13), Warring States (n=1), Spring and Autumn period (n=38), Han dynasty (n=26), Ming and Qing dynasties (n=13).

**Didong site (底董遗址)**

The Didong site is located in Lingbao City, Sanmenxia City, Henan Province, China[1]. It is an important early Yangshao culture settlement site covering an area of nearly 100,000 square meters. Excavations have revealed a rich variety of remains, including houses, kilns, and tombs. Notably, the discovery of rammed-earth structures indicates the mastery of this construction technology in the Central Plains no later than the early Yangshao period, providing a source for later architectural developments. In our study, 5 individuals from the Didong sites produced enough genomic data for analysis.

It should be noted that the archaeological background of individuals from Didong sites could be dated to early Yangshao culture period (~6000 years ago). However, the radiocarbon dating for three individuals with enough genomic data from Didong sites suggested that it could not be dated to 2000 years ago (i.e., individual 2006LD-M6, individual 06LDM8, and individual 2006LDM5 were directly radiocarbon dated to 2338-2157 cal BP. 2343-2157 cal BP, and 2319-2146 cal BP, respectively). We preferred the radiocarbon dating for these individuals when the discrepancy between their archaeological dating and radiocarbon dating data.

**Xiaowu site (****晓坞遗址)**

The Xiaowu site is located in Lingbao City, Henan Province, China[2]. This site, covering approximately 100,000 square meters, is primarily known for its early Yangshao culture (Dongzhuang type) remains. The key discoveries are two large, multi-person collective tombs featuring secondary burials. These finds reflect the close social ties and collective mortuary customs of the period and are crucial for studying early social organization, economy, and burial rituals in the region. In our study, 3 individuals from the early Yangshao cultural period produced enough genomic data for analysis. Sample 晓坞M2R7 was directly radiocarbon dated to 6289-6217 cal BP.

**Yangpingzhai site (阳平寨遗址)**

The Yangpingzhai site is located in Lingbao City, Henan Province, China[3]. Situated to the east of the Beiyangping site, the Yangpingzhai site is a component of the larger Zhudingyuan site complex. The human remains discovered at this particular site mainly date to the Longshan culture period. In our study, 9 individuals dating to the Longshan culture from Yangpingzhai sites produced enough genomic data for analysis. Sample 阳平寨R8 was directly radiocarbon dated to 4575-4425 cal BP.

**Nanwa site (****南洼遗址)**

The Nanwa site is located in Dengfeng City, Henan Province, China[4]. It is a large settlement site with an area of approximately 440,000 square meters. Its cultural deposits are mainly from the Erlitou culture period, with additional remains from the Yinxu, Spring and Autumn, and Tang-Song-Jin periods. The site provides important data for in-depth research on the settlement form of the Erlitou culture, and has important research value for studying the history before the Xia Dynasty, the Shang culture during the Yinxu period, and the origin of Chinese civilization. In our study, 4 individuals dating to the Shang Dynasty from the Nanwa sites produced enough genomic data for analysis. Sample YS-M19 was directly dated by radiocarbon dating to 3342-3174 cal BP.

**Shangshihe site (****上石河遗址)**

The Shangshihe site is located in Yima City, Henan Province, China[5]. It is a large and well-planned noble cemetery from the early to mid-Spring and Autumn Period. Based on bronze inscriptions, it is believed to be a burial ground for the exiled nobles and retainers of the State of Guo (虢国) after its fall in 655 BC. The site provides invaluable materials for studying the burial systems and customs of the Spring and Autumn period in western Henan and for conducting physical anthropology research on the people of Guo (虢人). In our study, 38 individuals dating to the Spring and Autumn period from the Shangshihe sites produced enough genomic data for analysis. Sample SSH M42 was directly dated by radiocarbon dating to 2732-2498 cal BP.

**Beiyangping site** **(北阳平遗址)**

The Beiyangping site is located in Lingbao City, Sanmenxia City, Henan Province, China[6]. It is a large settlement site with an area of about 720,000 square meters. In our study, 13 individuals dating to the Eastern Zhou dynasty from Beiyangping sites produced enough genomic data for analysis. Sample BYP-M24 was directly radiocarbon dated to 2359-2327 cal BP.

**Dongxiaoli Bailong site (****东小里白龙遗址)**

The Dongxiaoli Bailong site is located in Rongcheng County, Baoding City, Hebei Province, China[7]. It is a large commoner cemetery dating from the late Western Han to the late Eastern Han dynasty. As one of the few large Han Dynasty cemeteries in the Xiong'an New Area, the site is characterized by its large area, numerous well-organized tombs, and rich collection of artifacts, making it highly significant for studying the material life, burial customs, and social economy of the Han dynasty. In our study, 26 individuals dating to the Han dynasty from the Dongxiaoli Bailong sites produced enough genomic data for analysis. Sample DXL M22 was directly dated by radiocarbon dating to 2093-1927 cal BP.

**Wanhua site (****万花遗址)**

The Wanhua site is located in Wuzhi County, Henan Province, China[8]. It is a multi-period site with rich cultural deposits, most prominently from the Xia and Shang dynasties, but also including remains from the Han, Tang, Song, Yuan, Ming, and Qing periods. The site is crucial for understanding cultural interactions across the Qin River during the Xia period and the archaeological-cultural transformations in northern Henan during the Xia-Shang transition. Excavations also revealed a well-planned Qing dynasty cemetery, providing important clues about the funeral rites and social structure of the era. In our study, 13 individuals dating to the Ming and Qing Dynasties from the Wanhua sites. Sample WH M29 was directly dated by radiocarbon dating to 291-25 cal BP.

**Ancient DNA extraction and sequencing**

All experimental procedures were conducted in the specially designed ancient DNA cleanroom laboratory at Xiamen University [9–11]. We utilized 75% ethanol and 10% sodium hypochlorite (NaClO) to eliminate external contaminants. After treatment of surface abrasion and ultraviolet (UV) radiation, we employed a drill bit to obtain inner core bone powder ranging from 130 to 230 mg. For each 100 mg of bone powder, we added 1 ml of 0.5M EDTA and 1 μl of 20 mg/ml proteinase K, followed by incubation at 37°C on a shaker at 300 rpm for 20 hours. We used the MinElute System (Qiagen, Germany) to obtain concentrated DNA fragments. We prepared double-stranded libraries using the NEBNext® Ultra™ II DNA Library Prep Kit, paired with specially in-house designed sticky-end adaptors. We then sequenced the libraries on the DNBSEQ-T7 platform. We followed the protocol from David Reich's lab, employing Twist capture reagents for hybridization capture to enhance the acquisition of additional SNP loci[12]. Post-PCR clean-up throughout the entire experimental procedure was achieved using 1.8X Ampure beads (Beckman Coulter, USA) to eliminate non-target DNA fragments below 100 bp. We then sequenced the libraries on the DNBSEQ-T7 platform.

**Radiocarbon dating**

The preparation of bone/tooth samples involves standard acid-base-acid (ABA) procedure and collagen extraction. Firstly, bone/tooth samples are ultrasonicated in ultrapure water, dried, grinded and sieved to get the appropriately sized sample fraction (0.5–1 mm). Then samples are treated with 0.5M hydrochloric acid (~18 hrs), 0.1-0.2M sodium hydroxide (30 min-1h), and 0.5M hydrochloric acid (1 hr). Bone/tooth collagen gelatinization is performed in pH 3 solution at 70°C for 20 hrs. Gelatine solution is filtered using a cleaned filter and freeze-dried. The quality of collagen is monitored by carbon and nitrogen content in collagen, atomic C/N ratio determination and collagen yield. Samples are dated if the collagen yield is above 1% and the C:N ratio of the collagen is between 2.9 and 3.5. The samples that deviate from these ratios are deemed unsuitable for dating[13]. The pure collagen samples were combusted with CuO and silver in vacuo to CO2 at 900ºC for 2 hrs. The CO_2_ was purified, graphitized and measured on a compact 0.5 MeV NEC Accelerator Mass Spectrometry in the Guangzhou Institute of Geochemistry, Chinese Academy of Sciences[14]. All radiocarbon dates were modelled in OxCal[15] using the IntCal20[16] calibration curve.

**DNA sequence data processing**

Using AdapterRemoval (version 2.3.2)[17], we removed adapters from both read pairs, trimmed bases at 5'/3' termini with quality scores ≤20 and ambiguous bases (N) (--trimns --trimqualities --minquality 20), and collapsed forward and reverse reads (--collapse), and discarded reads shorter than 30 bp. Collapsed reads were aligned against the human reference genome hs37d5 (GRCh37 with decoy sequences) using the aln and samse modules in the Burrows-Wheeler Aligner (BWA) program (version 0.7.17-r1188)[18], with parameters ‘-l 1024’ (seeding disabled) and ‘-n 0.01’ (additional mismatches allowed). BAM files were sorted and indexed using Samtools (version 1.7) [19]before being utilized to remove PCR duplicates using dedup (version 0.12.8)[20]. BAM data were filtered with Samtools (version 1.7)[19] for a minimum Phred-scaled mapping quality score of 30.

**Data quality control**

We used a variety of methods to assess the ancient DNA's authenticity. First, we used mapDamage (version 2.2.1)[21] to compute the postmortem damage pattern. We determined if each library had >10% C-to-T misincorporations at 5' termini and >10% G-to-A misincorporations at 3' termini, as is expected for double-stranded libraries. Second, we estimated the contamination rate. To calculate the mtDNA contamination rate, (1) we used the schmutzi.pl module in schmutzi software[22] and the contaminant database developed for Eurasian samples from share/schmutzi/alleleFreqMT/eurasian/freqs; and (2) we followed the pipeline posted in <https://github.com/mnievesc/Ancient_mtDNA_Pipeline/blob/master/MT_DataAnalysis_Pipeline.sh> to conduct contamMix[23]-related analysis. The X chromosomal contamination rate was calculated for males using the contamination module in ANGSD software (version 0.910)[24]. We used the HapMap resources for CHB (Han Chinese in Beijing) provided by ANGSD software to define X chromosomal polymorphic sites. We focused our investigation on the non-recombining part of the X chromosome (X:5,000,000-154,900,000). The library was treated as contaminated when the mtDNA contamination was greater than 5%, except when nuclear DNA contamination was assessed by the X chromosome <5% for males.

**Biological sex determination**

An individual with one X chromosome and one Y chromosome was designated as "male," whereas an individual with two X chromosomes was designated as "female." Rx and Ry statistics[25,26] were computed to estimate the biological sex of the samples based on shotgun data. The biological sex of samples based on 1240K capture data was determined by the method described in Reference[27]. This method calculated the ratio of the alignments to chromosome Y to chromosome autosomes, divided by the expected value of the quantity based on the number of SNPs in the relevant target set. The depth of coverage was calculated by the depth module of Samtools (version 1.7)[19]. $\frac{\mathrm{YCov}}{\mathrm{autoCov}}$ is predicted to be approximately 0.5 for males and ~0 for females. Individuals with an observed $\frac{\mathrm{YCov}}{\mathrm{autoCov}}$ value of >0.3 are classified as males, whereas $\frac{\mathrm{YCov}}{\mathrm{autoCov}}$ value of <0.1 are classified as females, as described in Ref[28].

**Genotyping**

We used the trimBam module in BamUtil (version 1.0.15) [29] to mask 9 bp from both ends to reduce the bias caused by ancient DNA deamination. We randomly picked one high-quality base (--q 30 and --Q 30) as a pseudohaploid using pileupcaller (https://github.com/stschiff/sequenceTools).

**Estimation of genetic relatedness**

We used the default parameters of the READ software [30], KIN software[31], and ancIBD software[32] to estimate biological relatedness of each pair of individuals from the same archaeological sites.

We ran READ software[30] with default parameters based on the pseudo-genotyping files. We used unrelated individuals without having first-degree and second-degree kinships estimated by READ for subsequent genetic analyses.[33]

We introduced the hidden Markov-model based approach by KIN software[31] to test all the kinships within the second-degree. We ran KIN software on the Q30 bam files with 9 bp on both ends masked. It should be noted that KIN assumed the analyzed individuals originate from a single population; if a significant substructure exists in the actual population, the method may fail to infer relatedness accurately. In the population genetics analysis, we found that all genomes except for one genome from Yangpingzhai site showed within-group genetic homogeneity.

We also estimated IBD sharing using ancIBD software[32] based on the imputed diploid-genotyping data. Newly generated genomes from individuals with coverage above 0.1× on 1240k panel were imputed using software GLIMPSE2. We used 1000 Genome Project data as the haplotype reference panel (<https://ftp.1000genomes.ebi.ac.uk/vol1/ftp/release/20130502/>) to estimate genotype posterior probabilities and phased genotypes at bi-allelic SNP sites in the 1000 Genome data. For IBD analysis, we then restricted to variants in the 1240k SNP panel. To obtained the IBD sharing information, imputed files were merged by chromosomes and analyzed by the software ancIBD with suggested parameters, including genotype posterior probabilities higher than 0.99, gap maximum at 0.0075, and IBD blocks with more than 220 SNPs. In the ancIBD analysis, for pairs of individuals sharing more than two long IBD segments (greater than 20cM), we considered this pair of individuals to be related through first to sixth relatedness.

**Data merging**

We merged our newly generated samples with published genome-wide SNP data of present-day and ancient Eurasians using the *mergeit* function in EIGENSOFT software[34]. The information on co-analyzed ancient and modern genomes is listed in **Tables S1H and S1I**. Two datasets were used in our analysis: (1) We merged our data with a 1240k dataset curated by The Allen Ancient DNA Resource (AADR)[35], which covered ancient DNA data with maximum SNP sites (1,135,618). (2) We merged our data with the Human Origin (HO) dataset curated by The Allen Ancient DNA Resource (AADR)[35], which covered ancient DNA data with overlapped SNP sites between 1240k and HO SNP panel (593,120), as well as present-day Eurasians genotyped on Human Origin SNP chip.

**Principal Component Analysis (PCA)**

We ran PCA on the HO dataset using the smartpca (version 16000) algorithm in EIGENSOFT software[36] with the options "lsqproject: YES" and "numoutlieriter:0", and "shrinkmode: YES". We employed the "lsqproject: YES" option to project all ancient genomes onto the PC spaces calculated with the modern genomes.

***F* statistics**

We applied *qp3pop* (version 651) from the ADMIXTOOLS software[34] with the option "inbred: YES" to calculate outgroup *f*_3_ in the form of *f*_3_ (A, B; Yoruba). The outgroup *f*_3_ was used to quantify the shared genetic drift between A and B. We used *qpDstat* (version 980) from the ADMIXTOOLS software[34] with the option "f4mode: YES" to calculate *f*_4_ statistics in the form of *f*_4_ (Yoruba, A; B, C). The standard error (Std.err) was calculated with 5cM block jackknifing implemented in the ADMIXTOOLS software[34].

**Pairwise qpWave**

We conducted pairwise qpWave analysis using the qpWave program (version 600) in ADMIXTOOLS software [34]. We chose the following populations as the base outgroups: Mbuti.DG, CHG, Onge.DG, Iran_GanjDareh_N, Shandong_EN (a pool of Bianbian, Boshan, Xiaogao, and Xiaojingshan), Mongolia_N_East, Japan_Jomon, Kazakhstan_Eneolithic_Botai, Taiwan_Hanben, Upper_YR_LN, Shannan3k. Rank=0 models with p-value >0.01 suggested that the tested populations were genetically homogeneous compared to the base outgroups; in other words, one stream of ancestry from a set of outgroup populations could explain the ancestry of tested populations.

**Admixture modeling using qpAdm**

We modeled our genomes as an admixture of two source populations and estimated the proportions of ancestry using qpAdm (version 1000) in ADMIXTOOLS software [34], with settings “details: YES” and “allsnps: YES.” We chose the following populations as the base outgroups: Mbuti.DG, CHG, Onge.DG, Iran_GanjDareh_N, Shandong_EN (a pool of Bianbian, Boshan, Xiaogao, and Xiaojingshan), Mongolia_N_East, Japan_Jomon, Kazakhstan_Eneolithic_Botai, Ami.DG, Shannan3k. All the conditions listed below should be satisfied: (1) coefficient>0 and coefficient±Std.err>0; (2) p value>0.01; (3) p value for the nested model<0.05.

**References**

1.	Wei X. [Periodization of Yangshao Cultural Remains at Didong Site, and Issues Related](https://www.cnki.net/kns/Detail?sfield=fn&QueryID=31&CurRec=2&recid=&FileName=ZLBK201101010&DbName=CJFDHIS2&DbCode=CJFD&yx=&pr=&URLID=). Journal of the National Museum of China 2011;***1***:47-57 (in Chinese).

2.	Wei X. [The Trial Excavation of the Remains of Yangshao Culture at Xiaowu Site in Lingbao City,Henan](https://www.cnki.net/kns/Detail?sfield=fn&QueryID=5&CurRec=3&recid=&FileName=KAGU201112002&DbName=CJFD2011&DbCode=CJFD&yx=&pr=&URLID=). Archaeology 2011;***12***:3-22+113+97-98 (in Chinese).

3.	Wei X, Zhang X. A Geoarchaeological Study of the Neolithic Settlements and Their Developments in Zhudingyuan, Lingbao. Cultural Relics of Central Plains 2017;*6*:45-51 (in Chinese).

4.	Han G, Zhang J, Xu J. Brief report on the trial excavation of Nanwa site in Dengfeng, Henan in the spring of 2004. Cultural Relics of Central Plains 2006;*3*:4-12+22 (in Chinese).

5.	Yang H, Han H, Gao M, et al. A brief report on the excavation of the Spring and Autumn Tomb in Shangshihe, Yima, Henan Province. Cultural Relics of Central Plains 2019;*4*:4-18+2 (in Chinese).

6.	Wei X, Li S, Yang H, et al. Archaeological Exploration Report of Beiyangping Site in Lingbao City, Henan Province. Huaxia Archaeology 2020;*2*: 3-14+97+2 (in Chinese).

7.	Lu Y. A Study on Human Bones in the Han Dynasty Cemetery in Dongxiaoli, Xiong'an. *M.S. Thesis*. Zhengzhou University, 2022 (in Chinese).

8.	Liu Z. A brief report on the excavation of Shang Dynasty tombs at the Wanhua site in Wuzhi, Henan Province. Cultural Relics 2022;*12*:4-9+2 (in Chinese).

9.	Knapp M, Clarke AC, Horsburgh KA *et al.* Setting the stage – Building and working in an ancient DNA laboratory. *Annals of Anatomy - Anatomischer Anzeiger* 2012;194(1):3–6. https://doi.org/10.1016/j.aanat.2011.03.008.

10.	Llamas B, Valverde G, Fehren-Schmitz L *et al.* From the field to the laboratory: Controlling DNA contamination in human ancient DNA research in the high-throughput sequencing era. *STAR: Science & Technology of Archaeological Research* 2017;3(1):1–14. https://doi.org/10.1080/20548923.2016.1258824.

11.	Zhu K, He H, Tao L *et al.* Protocol for a comprehensive pipeline to study ancient human genomes. *STAR Protoc* 2024;5(2):102985. https://doi.org/10.1016/j.xpro.2024.102985.

12.	Rohland N, Mallick S, Mah M *et al.* Three assays for in-solution enrichment of ancient human DNA at more than a million SNPs. *Genome Res* 2022;32(11–12):2068–78. https://doi.org/10.1101/gr.276728.122.

13.	Van Klinken GJ. Bone Collagen Quality Indicators for Palaeodietary and Radiocarbon Measurements. *Journal of Archaeological Science* 1999;26(6):687–95. https://doi.org/10.1006/jasc.1998.0385.

14.	Zhu S, Ding P, Wang N *et al.* The compact AMS facility at Guangzhou Institute of Geochemistry, Chinese Academy of Sciences. *Nuclear Instruments and Methods in Physics Research Section B: Beam Interactions with Materials and Atoms* 2015;361:72–5. https://doi.org/10.1016/j.nimb.2015.06.040.

15.	Ramsey CB, Lee S. Recent and Planned Developments of the Program OxCal. *Radiocarbon* 2013;55(2):720–30. https://doi.org/10.1017/S0033822200057878.

16.	Reimer PJ, Austin WEN, Bard E *et al.* The IntCal20 Northern Hemisphere Radiocarbon Age Calibration Curve (0–55 cal kBP). *Radiocarbon* 2020;62(4):725–57. https://doi.org/10.1017/RDC.2020.41.

17.	Schubert M, Lindgreen S, Orlando L. AdapterRemoval v2: rapid adapter trimming, identification, and read merging. *BMC Res Notes* 2016;9(1):88. https://doi.org/10.1186/s13104-016-1900-2.

18.	Li H, Durbin R. Fast and accurate long-read alignment with Burrows–Wheeler transform. *Bioinformatics* 2010;26(5):589–95. https://doi.org/10.1093/bioinformatics/btp698.

19.	Li H, Handsaker B, Wysoker A *et al.* The Sequence Alignment/Map format and SAMtools. *Bioinformatics* 2009;25(16):2078–9. https://doi.org/10.1093/bioinformatics/btp352.

20.	Peltzer A, Jäger G, Herbig A *et al.* EAGER: efficient ancient genome reconstruction. *Genome Biol* 2016;17(1):60. https://doi.org/10.1186/s13059-016-0918-z.

21.	Ginolhac A, Rasmussen M, Gilbert MTP *et al.* mapDamage: testing for damage patterns in ancient DNA sequences. *Bioinformatics* 2011;27(15):2153–5. https://doi.org/10.1093/bioinformatics/btr347.

22.	Renaud G, Slon V, Duggan AT *et al.* Schmutzi: estimation of contamination and endogenous mitochondrial consensus calling for ancient DNA. *Genome Biol* 2015;16(1):224. https://doi.org/10.1186/s13059-015-0776-0.

23.	Fu Q, Mittnik A, Johnson PLF *et al.* A Revised Timescale for Human Evolution Based on Ancient Mitochondrial Genomes. *Current Biology* 2013;23(7):553–9. https://doi.org/10.1016/j.cub.2013.02.044.

24.	Korneliussen TS, Albrechtsen A, Nielsen R. ANGSD: Analysis of Next Generation Sequencing Data. *BMC Bioinformatics* 2014;15(1):356. https://doi.org/10.1186/s12859-014-0356-4.

25.	Skoglund P, Storå J, Götherström A *et al.* Accurate sex identification of ancient human remains using DNA shotgun sequencing. *Journal of Archaeological Science* 2013;40(12):4477–82. https://doi.org/10.1016/j.jas.2013.07.004.

26.	Mittnik A, Wang CC, Svoboda J *et al.* A Molecular Approach to the Sexing of the Triple Burial at the Upper Paleolithic Site of Dolní Věstonice. *PLoS ONE* 2016;11(10):e0163019. https://doi.org/10.1371/journal.pone.0163019.

27.	Fu Q, Posth C, Hajdinjak M *et al.* The genetic history of Ice Age Europe. *Nature* 2016;534(7606):200–5. https://doi.org/10.1038/nature17993.

28.	Lee J, Miller BK, Bayarsaikhan J *et al.* Genetic population structure of the Xiongnu Empire at imperial and local scales. *Sci Adv* 2023;9(15):eadf3904. https://doi.org/10.1126/sciadv.adf3904.

29.	Jun G, Wing MK, Abecasis GR *et al.* An efficient and scalable analysis framework for variant extraction and refinement from population-scale DNA sequence data. *Genome Res* 2015;25(6):918–25. https://doi.org/10.1101/gr.176552.114.

30.	Monroy Kuhn JM, Jakobsson M, Günther T. Estimating genetic kin relationships in prehistoric populations. *PLoS ONE* 2018;13(4):e0195491. https://doi.org/10.1371/journal.pone.0195491.

31.	Popli D, Peyrégne S, Peter BM. KIN: a method to infer relatedness from low-coverage ancient DNA. *Genome Biol* 2023;24(1):10. https://doi.org/10.1186/s13059-023-02847-7.

32.	Ringbauer H, Huang Y, Akbari A *et al.* Accurate detection of identity-by-descent segments in human ancient DNA. *Nat Genet* 2024;56(1):143–51. https://doi.org/10.1038/s41588-023-01582-w.

33.	Rubinacci S, Ribeiro DM, Hofmeister RJ *et al.* Efficient phasing and imputation of low-coverage sequencing data using large reference panels. *Nat Genet* 2021;53(1):120–6. https://doi.org/10.1038/s41588-020-00756-0.

34.	Patterson N, Moorjani P, Luo Y *et al.* Ancient Admixture in Human History. *Genetics* 2012;192(3):1065–93. https://doi.org/10/p4t.

35.	Mallick S, Micco A, Mah M *et al.* *The Allen Ancient DNA Resource (AADR): A Curated Compendium of Ancient Human Genomes*. Genomics, 2023.

36.	Patterson N, Price AL, Reich D. Population Structure and Eigenanalysis. *PLoS Genet* 2006;2(12):e190. https://doi.org/10.1371/journal.pgen.0020190.
